# Vanillin enones as selective inhibitors of the cancer associated carbonic anhydrase isoforms IX and XII. The out of the active site pocket for the design of selective inhibitors?

**DOI:** 10.1080/14756366.2021.1982933

**Published:** 2021-10-04

**Authors:** Leonardo E. Riafrecha, Macarena S. Le Pors, Martín J. Lavecchia, Silvia Bua, Claudiu T. Supuran, Pedro A. Colinas

**Affiliations:** a CEDECOR (UNLP-CICBA), CONICET, Departamento de Química, Facultad de Ciencias Exactas, Universidad Nacional de La Plata, La Plata, Argentina; b CEQUINOR (CONICET-UNLP) Facultad de Ciencias Exactas, Universidad Nacional de La Plata, La Plata, Argentina; c Laboratorio di Chimica Bioinorganica, Universitá degli Studi di Firenze, Florence, Italy; d NEUROFARBA Department, Section of Pharmaceutical Chemistry, Università degli Studi di Firenze, Florence, Italy

**Keywords:** Vanillin, carbonic anhydrase, enzyme inhibitors, molecular docking, enones

## Abstract

New *C*-glycosides and α,β-unsaturated ketones incorporating the 4-hydroxy-3-methoxyphenyl (vanillin) moiety as inhibitors of carbonic anhydrase (CA, EC 4.2.1.1) isoforms have been investigated. The inhibition profile of these compounds is presented against four human CA (hCA) isozymes, comprising hCAs I and II (cytosolic, ubiquitous enzymes) and hCAs IX and XII (tumour associated isozymes). Docking analysis of the inhibitors within the active sites of these enzymes has been performed and is discussed, showing that the observed selectivity could be explained in terms of an alternative pocket out of the CA active site where some of these compounds may bind. Several derivatives were identified as selective inhibitors of the tumour-associated hCA IX and XII. Their discovery might be a step in the strategy for finding an effective non-sulfonamide CA inhibitor useful in therapy/diagnosis of hypoxic tumours or other pathologies in which CA isoforms are involved.

## Graphical Abstract

**Figure:**
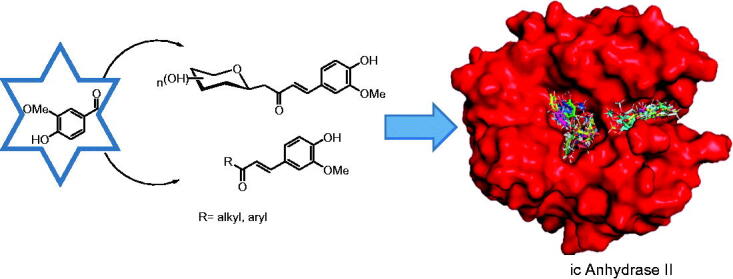

## 1. Introduction

Carbonic anhydrases (CAs, EC 4.2.1.1) are members of a great family of metalloproteins found in most organisms such as bacteria, archaea, fungi, protozoans, plants, algae, and vertebrates^1^. CAs catalyze one of the most important physiological reactions: the reversible hydration of carbon dioxide with the formation of a proton and bicarbonate^1^^,^^2^. Up to now eight genetically distinct CA families are known, the α-, β-, γ-, δ-, ζ-, η-, θ-, and ι-CAs. Mammals possess only α-CAs, while many pathogenic organisms such as bacteria and fungi encode enzymes belonging to several families, among which α-, β-, γ- and ι-CAs. Most of these enzymes contain a metal ion (usually Zn^2+^) in their active site, which is coordinated by three histidine residues and a water molecule/hydroxide ion in the α-CA class^1^^,^^2^, whereas the recently described ι-CAs seem to be devoid of metal ions and perform the catalysis by a diverse mechanism^3^.

In mammals, 16 different α-CA isoforms are known to date, with human CA isozymes hCA I and II (cytosolic forms) being the most widespread ones throughout the human body^1^. The disregulated CA activity is linked with numerous pathological states^1^. In recent years, the connection between the overexpression of transmembrane isoforms hCA IX and XII with cancer progression has been investigated in detail and clarified^4^. These isozymes are overexpressed in solid tumours as a consequence of the hypoxia-inducible factor-1 (HIF-1) signalling cascade, being less expressed in several normal tissues^4^^,^^5^. Thus selective inhibition of tumour-associated isoforms hCA IX and XII (over the off-target hCA I and II) is a great promise for the use of CA inhibitors in the cancer therapy/diagnosis^4^^,^^5^.

The intake of protective factors for fortifying the natural bodýs defense capacity to reduce the risk of cancer is an approach called chemoprevention. Recently natural products containing phenols, have been recognised as cancer chemopreventive agents^6–8^. Ginger (*Zingiber officinale*) is one of the most widely consumed spices in the world^6^^,^^7^ Ginger contains several phenolic compounds possessing antimicrobial and analgesic activity, such as 6-gingerol and 6-paradol that incorporate vanillin (4-hydroxy-3-methoxyphenyl) moiety in their molecules. Related 6-dehydroparadols have shown antitumor-promoting activity in mouse skin carcinogenesis^9^. 6-Shogaol, another vanillin derivative found in ginger, suppresses the proliferation of non-small cell lung cancer^9^. Phenol derivatives, including some natural products, have also been studied as CA inhibitors (CAIs)^10–13^.

Several phenolic derivatives share a common moiety: an α,β-unsaturated carbonyl group, or enone. Incorporation of this functionality into a given molecular structure has been shown to increase antitumor activity^14^. In that sense, the introduction of an enone moiety into a triterpenoid skeleton had enhanced the cytotoxic activity against human breast cancer cells^15^.

Our group has developed several selective CAIs by the attachment of *C*-glycosidic enones to carbohydrates^16^^.^ The use of carbohydrate moieties could induce the desired physicochemical properties such as water solubility, lower permeability in organs in which the enzyme is also present, etc^5^. Although several pharmacophores have thoroughly been investigated in the design of sugar-based CAIs^17^, there are still several functionalities that have been only poorly studied such as the vanillin moiety. To the best of our knowledge no alkyl nor aryl enones of vanillin have been studied so far as CAIs^18^.

## 2. Materials and methods

### 2.1. Materials and apparatus

All starting materials and reagents were purchased from commercial suppliers. Reactions were monitored by TLC and TLC plates visualised with short wave UV fluorescence (*k* = 254 nm), sulphuric acid stain (5% H_2_SO_4_ in methanol). Silica gel flash chromatography was performed using silica gel 60 Å (230–400 mesh). All melting points are uncorrected. ^1^H and ^13 ^C NMR spectra were recorded on a Bruker 600 (600 and 151 MHz, respectively). Chemical shifts were measured in ppm and coupling constants in Hz. High-resolution mass spectra were recorded using electrospray as the ionisation technique in positive ion or negative ion modes as stated. All MS analysis samples were prepared as solutions in methanol.

### 2.2. General procedure for the synthesis of compounds 1 and 2

A mixture of β-*C*-glucopyranosyl or β-*C*-galactopyranosyl ketone (1 mmol), 4-hydroxy-3-methoxybenzaldehyde (vanillin) (1.2 mmol), and L-proline (0.15 mmol)-TEA (0.3 mmol) in 3 ml of methanol was stirred at reflux for 48–96 h. The endpoint of the reaction was monitored by TLC. The solvent was evaporated under reduced pressure and the product was purified by column chromatography (eluant 8:2 DCM-MeOH) to afford pure material by 1H NMR and 13 C NMR spectroscopy. Yields 35–45%.

### 2.3. General producer for the synthesis of compounds 3–7

A mixture of ketone (1 mmol) and 4-hydroxy-3-methoxybenzaldehyde (vanillin) (1 mmol) in 2 ml of anhydrous methanol has been added to the catalyst (see Table 1). The reaction was stirred at the corresponding temperature until starting material was consumed as evidenced by TLC, followed by dilution with ice-cold water, acidification with cold 1 M HCl, and extraction with DCM. The solvent was evaporated under reduced pressure. The product was purified by column chromatography (eluant 9:1 DCM-MeOH) to afford pure material by ^1^H NMR and ^13 ^C NMR spectroscopy. Yields 40–79%.

**Table 1.** Synthesis of vanillin enones **1**–**7.**

Ketone	Catalyst	Temperature	Reaction time (h)	Product	Yield (%)
*C*-glucosyl	Et_3_N/L-Pro	Reflux	48	1	45
*C*-galactosyl	Et_3_N/L-Pro	Reflux	96	2	35
Acetone	NaOH	40 °C	48	3	63
Butanone	Pyrrolidine	Rt	48	4	79
Methyl isobutyl ketone	Pyrrolidine	Rt	72	5	76
Acetophenone	Et_3_N/L-Pro	Reflux	44	6	70
*p*-Bromoacetophenone	Et_3_N/L-Pro	Reflux	72	7	40

**1-(β-D-glucopyranosyl)-4–(4-hydroxy-3-methoxyphenyl)but-3-en-2-one (1).** Sticky yellow solid. ^1^H NMR (600 MHz, DMSO) δ 9.62 (s, 1H, OH), 7.50 (d, 1H, *J* = 16.1 Hz, H-4), 7.31 (d, 1H, *J* = 2.0 Hz, ArH), 7.14 (dd, 1H, *J* = 8.2, 2.0 Hz, ArH), 6.82 (d, 1H, *J* = 1.6 Hz, ArH), 6.80 (d, 1H, *J* = 9.6 Hz, H-3), 5.03 (d, 1H, *J* = 5.7 Hz, OH), 4.91 (d, 1H, *J* = 4.6 Hz, OH), 4.85 (d, 1H, *J* = 4.8 Hz, OH), 4.34 (t, 1H, *J* = 5.7 Hz, OH), 3.83 (s, 3H, CH_3_O), 3.62 (dd, 1H, *J* = 9.2, 2.5 Hz, H-4′), 3.59 (m, 1H, H-6’b), 3.39 (dt, 1H, *J* = 11.2, 5.4 Hz, H-6’a), 3.17 (t, 1H, *J* = 7.7 Hz, H-1′), 3.06 (m, 2H, H-2′, H-5′), 2.95 (dt, 1H, *J* = 9.1, 4.8 Hz, H-1b), 2.91 (dd, 1H, *J* = 16.0, 2.6 Hz, H-3′), 2.77 (m, 1H, H-1a).^13^C NMR (151 MHz, DMSO) δ 198.23 (C-2), 149.81 (ArC), 148.39 (ArC), 143.28 (C-4), 126.43 (ArC), 124.32 (C-3), 123.77 (ArC), 116.07 (ArC), 111.80 (ArC), 81.14 (C-5′), 78.61 (C-1′), 76.38 (C-4′), 74.08 (C-3′), 70.79 (C-2′), 61.63 (C-6′), 56.15 (CH_3_O), 43.66 (C-1). HMRS *m/z*: calcd for C_17_H_22_O_8_, 354.1315; found, 352.1134.

**1-(β-D-galactopyranosyl)-4–(4-hydroxy-3-methoxyphenyl)but-3-en-2-one (2).** Sticky red solid. ^1^H NMR (600 MHz, DMSO) δ 9.04 (s, 1H, OH), 7.54 (d, 1H, *J* = 16.1 Hz, H-4), 6.97 (dd, 1H, *J* = 8.8, 1.9 Hz, ArH), 6.85 (dd, 1H, *J* = 8.2, 2.0 Hz, ArH), 6.82 (d, 1H, *J* = 8.1 Hz, ArH), 6.75 (d, 1H, *J* = 8.1 Hz, H-3), 5.42 (m, 1H, OH), 5.02 (d, 1H, *J* = 5.6 Hz, OH), 4.87 (d, 1H, *J* = 5.9 Hz, OH), 4.11 (q, 1H, *J* = 5.3 Hz, H-5′), 4.06 (m, 1H,H-2′), 3.93 (m, 1H, H-6’a), 3.81 (s, 1H, OH), 3.76 (s, 3H, CH_3_O), 3.58 (dd, 1H, *J* = 9.2, 3.0 Hz, H-1′), 3.44 (m, 1H, H-4′), 3.42 (m, 1H, H-6’b), 3.29 (dd, 1H, *J* = 9.3, 5.7 Hz, H-3′), 3.17 (d, 2H, *J* = 5.2 Hz, H-1a, H-1b).^13^C NMR (151 MHz, DMSO) δ 207.31 (C-2), 147.53 (ArC), 147.28 (ArC), 143.37 (C-4), 119.49 (ArC), 115.26 (C-3), 111.12 (ArC), 106.78 (ArC), 100.46 (ArC), 77.01 (C-5′), 76.43 (C-1′), 73.67 (C-4′), 70.35 (C-3′), 69.61 (C-2′), 69.25 (C-6′), 56.18 (CH_3_O), 46.69 (C-1). HMRS *m/z*: calcd for C_17_H_22_O_8_, 354.1315; found, 352.1130.

**(*E*)-4–(4-Hydroxy-3-methoxyphenyl)but-3-en-2-one (3**). White solid. Mp 122–122.5 °C. ^1^H NMR (600 MHz, CDCl_3_) δ 9.85 (sa, 1H, OH), 7.47 (d, 1H, *J* = 16.2 Hz,H-4), 7.10 (m, 3H, ArH), 6.95 (d, 1H, *J* = 8.2 Hz, ArH), 6.61 (d, 1H, *J* = 16.2 Hz, H-3), 3.95 (s, 3H, CH_3_O), 2.39 (s, 3H, CH_3_).^13^C NMR (151 MHz, CDCl_3_) δ 198.44 (C-2), 148.28 (ArC), 146.89 (ArC), 143.77 (C-4), 126.92 (ArC), 124.99 (C-3), 123.52 (ArC), 114.83 (ArC), 109.33(ArC), 55.97(**C**H_3_O), 27.31(CH_3_). HMRS *m/z*: calcd for C_11_H_12_O_3_, 192.0786; found, 192.0784.

**(*E*)-1–(4-Hydroxy-3-methoxyphenyl)pent-1-en-3-one (4)**. Yellow solid. Mp 81–82 °C. ^1^H NMR (600 MHz, CDCl_3_) δ 7.51 (d, 1H, *J* = 16.1 Hz,H-1), 7.09 (m, 2H, ArH), 6.94 (d, 1H, *J* = 8.1 Hz, H-2), 6.62 (d, 1H, *J* = 16.1 Hz, ArH), 3.94 (s, 3H,CH_3_O), 2.70 (q, 2H, *J* = 7.3 Hz,H-4), 1.18 (t, 3H, *J* = 7.3 Hz,H-5).^13^C NMR (151 MHz, CDCl_3_) δ 201.10 (C-3), 148.15 (ArC), 146.88 (ArC), 142.57 (C-1), 127.07 (ArC), 123.81 (C-2), 123.34 (ArC), 114.83 (ArC), 109.47 (ArC), 55.97 (**C**H_3_O), 33.76 (C-4), 8.39 (C-5). HMRS *m/z*: calcd for C_12_H_14_O_3_, 206.0943; found, 206.0950.

**(*E*)-1–(4-Hydroxy-3-methoxyphenyl)-5-methylhex-1-en-3-one (5).** Sticky dark red solid. ^1^H NMR (600 MHz, CDCl_3_) δ 9.85 (d, 1H, *J* = 1.6 Hz, OH), 7.49 (d, 1H, *J* = 16.1 Hz, H-1), 7.12 (dd, 1H, *J* = 8.3, 1.8 Hz, ArH), 7.07 (d, 1H, *J* = 2.2 Hz, ArH), 6.95 (d, 1H, *J* = 8.1 Hz, H-2), 6.62 (d, 1H, *J* = 16.2 Hz, ArH), 3.95 (s, 3H, CH_3_O), 2.54 (d, 2H, *J* = 7.2 Hz, H-4), 2.25 (m, 1H, H-5), 1.00 (d, 6H, *J* = 6.6 Hz, H-6, H-7).^13^C NMR (151 MHz, CDCl_3_) δ 200.42 (C-3), 148.16 (ArC), 146.86 (ArC), 142.73 (C-1), 127.09 (ArC), 124.48 (C-2), 123.38 (ArC), 114.82 (ArC), 109.47 (ArC), 55.97 (**C**H_3_O), 49.69 (C-4), 25.39 (C-5), 22.74 (C-6,C-7). HMRS *m/z*: calcd for C_14_H_18_O_3_, 234.1256; found, 234.1260.

**(*E*)-3–(4-Hydroxy-3-methoxyphenyl)-1-phenylprop-2-en-1-one (6).** Sticky yellow solid. ^1^H NMR (600 MHz, CDCl_3_) δ 9.84 (sa, 1H, *J* = 1.4 Hz, OH), 7.77 (d, 1H*, J* = 15.6 Hz, H-3), 7.59 (m, 1H, ArH), 7.52 (d, 1H, *J* = 7.3 Hz, ArH), 7.44 (dd, 1H, *J* = 6.0, 1.7 Hz, ArH), 7.40 (d, 1H, *J* = 15.6 Hz,H-2), 7.28 (d, 1H, *J* = 1.4 Hz, ArH), 7.23 (dd, 1H, *J* = 8.3, 1.8 Hz, ArH), 7.15 (d, 1H, *J* = 1.8 Hz, ArH), 7.06 (dd, 1H, *J* = 8.5, 1.5 Hz, ArH), 6.98 (dd, 1H, *J* = 8.2, 1.4 Hz, ArH), 3.98 (s, 3H, *J* = 1.5 Hz,CH_3_O).^13^C NMR (151 MHz, CDCl_3_) δ 190.91 (C-1), 151.72 (ArC), 148.35 (ArC), 147.18 (C-3), 145.30 (C-2), 138.49 (ArC), 132.58 (ArC), 129.88 (ArC), 128.57 (ArC), 128.44 (ArC), 127.53 (ArC), 123.42 (ArC), 119.82 (C-3), 114.41 (ArC), 108.81 (ArC), 56.12 (**C**H_3_O). HMRS *m/z*: calcd for C_16_H_14_O_3_, 254.0943; found, 254.0948.

**(*E*)-1–(4-Bromophenyl)-3–(4-hydroxy-3-methoxyphenyl)prop-2-en-1-one (7).** Sticky yellow solid. ^1^H NMR (600 MHz, CDCl_3_) δ 9.85 (s, 1H, OH), 7.89 (d, 2H, *J* = 8.5 Hz, ArH), 7.77 (d, 1H, *J* = 15.6 Hz, H-3), 7.66 (d, 2H, *J* = 8.6 Hz, ArH), 7.33 (d, 1H, *J* = 15.6 Hz, H-2), 7.24 (dd, 1H, *J* = 8.2, 2.0 Hz, ArH), 7.14 (d, 1H, *J* = 1.9 Hz, ArH), 6.98 (d, 1H, *J* = 8.2 Hz, ArH), 3.99 (s, 3H, CH_3_O). ^13 ^C NMR (151 MHz, CDCl_3_) δ 189.43 (C-1), 148.54 (ArC), 146.87 (ArC), 145.74 (C-3), 137.27 (ArC), 131.84 (2 x ArC), 129.94 (2 x ArC), 127.56 (ArC), 127.33 (ArC), 123.48 (ArC), 119.27 (C-2), 114.95 (ArC), 110.18 (ArC), 56.06 (**C**H_3_O). HMRS *m/z*: calcd for C_16_H_13_BrO_3_, 332.0048; found, 332.0061.

### 2.4. CA inhibition studies

An Applied Photophysics stopped-flow instrument has been used for assaying the CA catalysed CO_2_ hydration activity as reported by Khalifah^19^. Phenol red (at a concentration of 0.02 mM) has been used as an indicator, working at the absorbance máximum of 557 nm, with 20 mM Hepes (pH 7.5) as a buffer, and 20 mM Na_2_SO_4_ (for maintaining constant the ionic strength), following the initial rates of the CA-catalysed CO_2_ hydration reaction for a period of 10–100 s. The CO_2_ concentrations ranged from 1.7 to 17 mM for the determination of the kinetic parameters and inhibition constants. For each inhibitor, at least six traces of the initial 5–10% of the reaction have been used for determining the initial velocity. The uncatalyzed rates were determined in the same manner and subtracted from the total observed rates. Stock solutions of inhibitor (0.1 mM) were prepared in distilled–deionized water and dilutions up to 0.01 nM were done thereafter with distilled–deionized water. Inhibitor and enzyme solutions were preincubated together for 15 min at room temperature prior to assay, in order to allow for the formation of the E-I complex. The inhibition constants were obtained by non-linear least-squares methods using PRISM 3, and the Cheng-Prussoff equation as reported earlier^20^ and represent the mean from at least three different determinations.

### 2.5. Computational methods

#### 2.5.1. Preparation of the molecular systems

The simulations were based on X-ray crystal structures of human carbonic anhydrases (CA I: PDB ID 2FW4, CA II: PDB ID 3KS3, CA IX: PDB ID 6FE2, CA XII: PDB ID 1JCZ). These structures were selected from the Protein Data Bank^21^, based on resolution, validation parameters, and missing residues.

The preparation of proteins was done with Chimaera^22^. Water molecules and other ligands were removed. All Asp and Glu residues were considered to have a negative charge and all the Arg and Lys residues were considered to have a positive charge. Hydrogens were added following the hydrogen-bonding pattern. Ligand structures were built with Avogadro^23^ and then optimised using the PM6 semiempirical method, implemented in OpenMOPAC^24^. The solvent effect was considered using the COSMO implicit model^25^, with a dielectric constant value of 78.4, corresponding to an aqueous medium. The geometry optimisation termination criteria were set to 0.1 kcal/mol/Å gradient norm requirement.

#### 2.5.2. Molecular docking

Molecular docking was carried out to find and score protein-ligand binding poses on carbonic anhydrase structures with Smina with Vina as scoring function^26^. Protein and ligand PDBQT files were prepared with AutoDockTools software^27^. To unify the box set and simplify the analysis of results, chains A of 2FW4, 6FE2, 1JCZ structures were aligned to 3KS3 using Pymol^28^. A docking box with size 27.75 Å × 27.00 Å × 28.50 Å was centred on the catalytic binding site. The ligands were docked using a flexible-ligand/rigid-receptor approach. The exhaustiveness value was increased to 20.

In order to have alternative poses, DOCK 6.8^29^ was also used with grid score and flexible ligands and applying a subsequent minimisation. The spheres selected where the grid is calculated were the ones that fit inside the box used with Smina. Other parameters were set to their default values.

The post-docking analysis included visualisation of the ligand-receptor complexes with Pymol to analyse the potential interactions with the amino acid.

#### 2.5.3. Binding energy estimated by semiempirical method

In order to obtain greater precision in the characterisation of the interaction of ligands with carbonic anhydrase isozymes, a calculation scheme based on the semi-empirical PM6^30^ was adopted, employing OpenMOPAC ^24^. It was decided to implement a quantum method in view of the presence of Zn^2+^ in the carbonic anhydrase, which makes it somewhat difficult to deal with molecular mechanical force fields, in particular, to estimate binding energy.

Due to the considerable size of the system, the MOZYME^31^^,^^32^ approach was used, which has already been used to improve docking scoring functions or estimate binding energies^33–35^. The solvent effect was also considered using the COSMO implicit model.

A molecular optimisation of all the poses obtained by docking was performed, both on the complexes, as well as on the ligands and the carbonic anhydrase separately. Then, binding enthalpies were calculated as the heat of formation differences (ΔH_bind_ = Δ_f_H_complex_ - Δ_f_H_ligand_ - Δ_f_H_CA_).

#### 2.5.4. Molecular dynamics

MD simulations were performed on poses resulting from molecular docking for CA I and CAII. The system, CAs, and ligands have a positive net charge, so chloride anions were added as counterions with the Leap module to achieve electroneutrality. The neutralised ligand/CA complexes were immersed in a box of TIP3P waters which extended up to 15 Å from the solute. CAs were described using the Amber14SB force field^36^. The ligand was described using the Generalised Amber Force Field^37^ with charges derived from RESP, which were calculated with the Antechamber module. Leap and Antechamber are included in the package AmberTools 20.0^38^. Zn^2+^ cation, neighbouring residues and a water molecule were modelled with MCPB.py^39^, also included in AmberTools 20.0.

All MD simulations were run using the NAMD 2.13 software^40^. The van der Waals interaction cut-off distances were set at 12 Å and long-range electrostatic forces were computed using the particle mesh Ewald summation method with a grid size set to 1.0 Å. The 1–4 contributions were multiplied by a factor of 0.83 to match the AMBER force field requirements. The system was subjected to 100000 minimisation steps, heating from 0 to 300 K in 30 ps, and 10 ns of equilibration/production simulation. This trajectory extension was chosen to achieve a balance between the number of simulations to run with respect to sample at equilibrium conditions, and also considering that the CAs quickly reach the equilibrium condition. For all equilibration/production simulations, constant temperature (300 K) was maintained using Langevin dynamics with a damping coefficient of 5 ps^−1^, while pressure was kept constant at 1 atm through the Nosé-Hoover Langevin piston method with a decay period of 200 fs and a damping time constant of 100 fs. A time step of 1 fs was used along with molecular mechanics. Bonds involving hydrogen atoms of waters were constrained using the SHAKE algorithm. RMSD values were depicted to determine the convergence and stability of simulations. (See Electronic Supplementary Material, Figures S2 and S3)

#### 2.5.5. Binding energy estimated by MM/GBSA

Binding free energies of ligands with CAs were computed using the MM-GB/SA method, where the binding free energy is calculated as the difference between the bound and unbound states of protein and ligand^41^^,^^42^.

The solvation free energy was calculated using the generalised Born (GB) model^43^ implemented in MMPBSA.py module^44^, igb = 5 as the selected model. The hydrophobic contribution was determined using the solvent-accessible surface area. The CAs–ligands binding free energies were calculated using a single trajectory (for ligand, protein, and complex) based on 500 snapshots taken from the last 5 ns portion of the MD simulation trajectories. Entropies were estimated using quasi-harmonic approximation.

To obtain the detailed representation of interactions, free energy decomposition analysis was employed to decompose the total binding free energies into ligand–amino acid pairs. These calculations were performed using a pairwise energy decomposition scheme (idecomp option 3) also with the MMPBSA.py module.

## 3. Results and discussion

### 3.1. Chemistry and CA inhibition

**Scheme 1.**
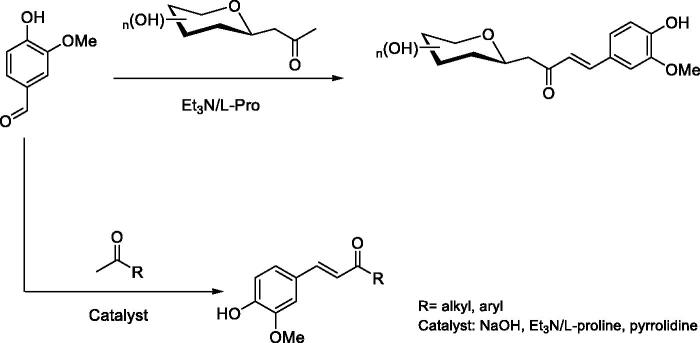
Preparation of vanillin enones **1–7** discussed in the paper

**Figure 1.**
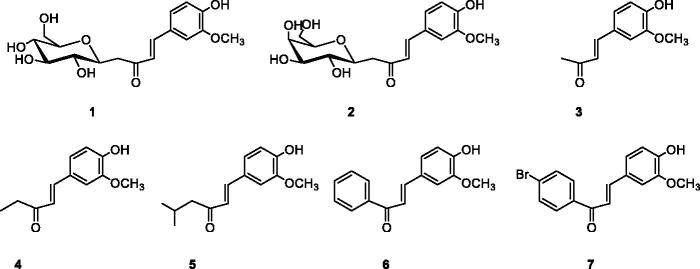
Structure of compounds **1–7**.

The enones **1**–**7** (Figure 1) have been prepared by aldol condensation of vanillin with the appropriate alkyl, aryl, or glycosidic methyl ketones (Scheme 1). Knoevenagel reaction of D-glucose or D-galactose with pentane-2,4-dione in the presence of aqueous sodium bicarbonate at 90 °C afforded the β-D-glycosyl-propan-2-ones in 50% and 54% yields, respectively. It should be noted that higher yields were described in the literature^45^, but the products had not been purified as in our report.

*C*-glycosides **1**–**2** have been prepared by reaction of β-*C*-glucosyl or β-*C*-galactosylketone with vanillin in the presence of L-proline/Et_3_N with moderate yields (Table 1). Next, we studied the aldol condensation of aliphatic or aromatic ketones and vanillin with different catalysts. The best reaction conditions found in the synthesis of the α,β-unsaturated ketones are shown in Table 1. Enones **1**–**7** were successfully purified by flash chromatography. The ^1^H NMR, ^13 ^C NMR, 2 D COSY and HSQC were in full agreement with their structures (see Supplementary information). The large coupling constant (*J* ≈ 16 Hz) between the two olefinic protons, was consistent with the *E* configuration of the double bond. In enones, **1**–**2** the large coupling constant (*J* = 9.4 Hz) between H-10 and H-20 indicated a diaxial relationship and thus confirmed the β-configuration.

The inhibitory activity of compounds **1–7** against cytosolic isoforms hCA I and II as well as the membrane-associated isoforms hCA IX and XII was assayed by using a stopped-flow assay method and the results are shown in Table 2. A number of structure-activity relationships were identified in this study and are summarised as follows.

**Table 2.** Inhibition of hCA I, II, IX, XII with vanillin enones **1**–**7**.^a^^,b^

	Ki (µM)^b^				Selectivity ratio			
Cmpd	hCA I	hCA II	hCA IX	hCA XII	I/IX	I/XII	II/IX	II/XII
**1**	71.6	>100	30.8	4.8	2.3	15.0	>3.2	>21
**2**	80.5	>100	20.8	2.5	3.9	32.2	>4.8	>40
**3**	86.3	>100	24.0	3.2	3.6	27.0	>4.2	>31
**4**	>100	>100	32.0	2.5	>3.1	>40	>3.1	>40
**5**	>100	>100	26.3	1.7	>3.8	>59	>3.8	>59
**6**	76.3	96.4	29.1	0.63	2.6	121.1	3.3	153.0
**7**	92.6	82.0	>100	6.4	<0.9	14.5	<0.8	12.8
**AAZ**	0.25	0.012	0.025	0.006	0.03	41.6	0.001	2

^a^All CAs are recombinant enzymes obtained in the authors’ laboratory as reported earlier^46–50^.

^b^Errors in the range of 5–10% of the reported value, from three different determinations.

#### 3.1.1. Off-target CA isozymes

- *C*-glycosides **1** and **2** were micromolar inhibitors of hCA I. A similar trend was found for the aryl derivatives **6** and **7** and the methyl one **3**. The alkyl derivatives **4** and **5** were very poor inhibitors of hCA I. It is of great interest to relate the behaviour of these compounds towards hCA I and it can be concluded that attachment of a voluminous scaffold such as the carbohydrate one, to the enone functionality, does not lead to a decrease in the inhibitory potency of these compounds against hCA I.
- Vanillin derivatives showed an interesting inhibition profile against hCA II. It should be noted that the *C*-glycosidic derivatives **1-2** and the alkyl enones **3-5** were very poor inhibitors of hCA II. On the other hand, the aromatic derivatives **6-7** were inhibitors acting in the micromolar range. However, being a ubiquitous, housekeeping isoform, this may not be a valuable property in another context if compounds targeting other isoforms (such as hCA IX and XII) should possess activity against hCA II. However, it is interesting to note that compounds **1-5** showed poor inhibition against hCA II while retaining effective inhibition against hCA IX and XII.

#### 3.1.2. Cancer-associated CA isozymes

- The tumour-associated target isoform hCA IX was inhibited in the submicromolar range by all enones except compound **7**, which was a poor inhibitor of this isozyme. The best inhibitor was the *C*-galactoside **2**, which weakly inhibited hCA II too.
- The second tumour-associated isoform, hCA XII, was the most inhibited isoform by all vanillin derivatives. The phenyl enone **6** was the most active compound in the series.
- Selectivity for inhibiting the tumour-associated isoforms (hCA IX and XII) over the widespread cytosolic forms (hCA I and II) is a key issue when designing CAIs. As can be in Table 2 several compounds showed better activity profiles against hCA IX and XII over I and II which is highly desirable when only the tumour-associated isoforms would be targeted. It was observed that enones **1-5**, which showed very good inhibition of isoforms IX and XII were also shown to be highly selective. The most effective hCA IX inhibitor **2** showed an excellent selectivity ratio over hCA II. Only aryl enones **6-7** showed almost no selectivity and are not useful in the design of selective inhibitors.

### 3.2. Molecular docking

**Figure 2.**
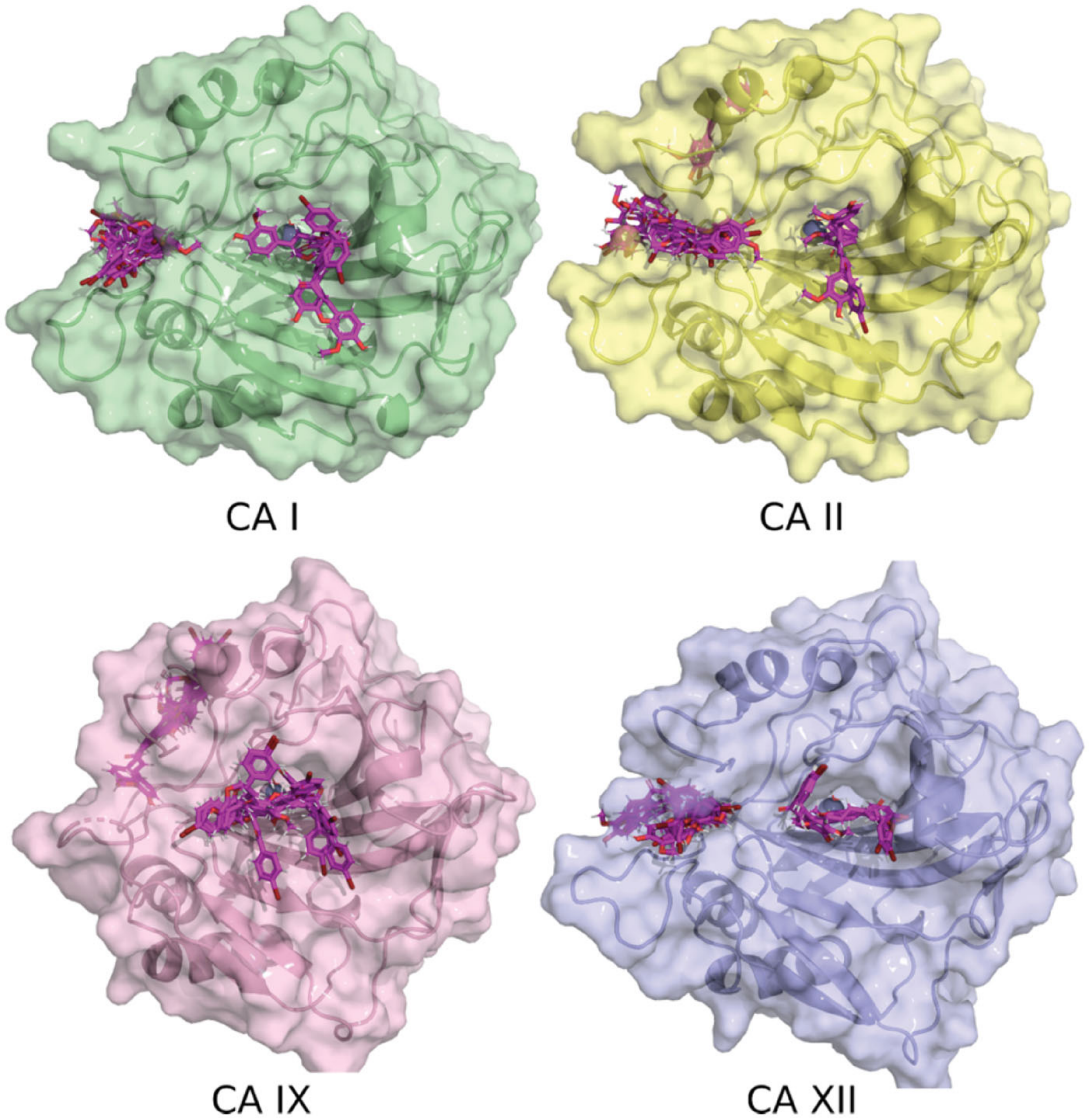
Binding poses of **7** to four CA isozymes obtained through molecular docking using Smina program. The ligand poses can be seen inside the catalytic site. In addition, some poses show the inhibitor bound in the adjacent pocket (oriented to the left) to the entrance at the active site cavity in the case of isoforms CA I, II, and XII.

It was observed, from the poses generated using molecular docking, that the various ligands presented different binding patterns for the analysed CA isozymes. Thus, in addition to ligand poses within the catalytic active site, the location of some poses indicates that the inhibitors might bind at an adjacent pocket, at the entrance of the active site, Figure 2. This site was previously described for hCA II as an alternative site for inhibitor interaction, and only one carboxylic acid derivative was observed to bind in it by means of X-ray crystallography^51^. The hCA I active site is aligned among others by residues Asp1, Gly3, Tyr4, Asp5, Asn8, His61, Lys167, Ser228, and Met238. In the case of hCA II, the site is integrated, in addition to the previously reported residues by Gly6, Tyr7, Gly8, Asn11, His64, Phe231, Asn232, and Glu239, by Trp5, Gly63, and Lys170^51^.

The hCA XII pocket is characterised by the presence of residues Lys1, Lys166, and Arg238, which are bulkier than the equivalent residues in hCA I and hCA II. In the case of hCA IX (PDB Id 6FE2), this pocket is absent and some docking poses are located close to this, on the rear face of the structure, as shown in Figure 2.

#### 3.2.1. Calculated binding enthalpies

In order to obtain more precise energies of the interaction with CAs, semi-empirical calculations were performed on the obtained docking poses. The binding enthalpies calculated with PM6 and the MOZYME approach are listed in Table S1. The energies were classified with a Python script according to the location of ligands, at the catalytic site or in the adjacent pocket, reporting the most stable energy value for each category when it was observed. In most cases, it was found that the poses in the alternative pocket have a lower (more favourable) binding enthalpy with respect to the location within the catalytic site. The exception to this observation is the CA IX isoform, where poses located at the catalytic site were more favourable than the out of the active site binding^51^.

#### 3.2.2. Molecular dynamics and binding free energies

Although the ligand structures and nearby residues are optimised when applying the semiempirical methodology, this represents a "snapshot" of the enzyme-inhibitor complex. To analyse the "film" of the system, molecular dynamics simulations and subsequent estimation of the binding energy on the hCA I and hCA II isozymes were thereafter performed. The calculated binding free energies including the entropy as quasi-harmonic approximation are shown in Table 3. It was observed in most cases that the interaction in the alternative pocket was more stable than poses placing the inhibitor inside the catalytic cavity with the methoxyphenol derivative close to the Zn^2+^ cation, this situation being more favourable than the poses localising the inhibitor inside the catalytic cavity with the outward-facing methoxyphenol.

**Table 3.** Binding free energies (kcal mol^−1^) of ligands with hCA I and hCA II calculated with MM-GBSA.

Ligand	Pose	hCA I	hCA II
1	Down	41.0	33.4
	Up	–	–
	Pocket	25.6	25.9
2	Down	33.3	7.0
	Up	–	–
	Pocket	32.3	28.0
3	Down	20.1	24.9
	Up	29.3	20.8
	Pocket	19.7	Unstable
4	Down	Unstable	Unstable
	Up	35.0	Unstable
	Pocket	22.5	31.3
5	Down	32.9	32.8
	Up	Unstable	–
	Pocket	27.7	31.3
6	Down	Unstable	30.3
	Up	Unstable	–
	Pocket	18.6	17.0
7	Down	37.7	23.8
	Up	Unstable	–
	Pocket	18.8	21.5

A correlation was observed for the compounds that presented a more favourable calculated binding energy with respect to those that showed better inhibitory capacity in the experimental assays (compounds **3**, **6**, and **7** with isoform hCA I, and compounds **6** and **7** with isoform hCA II). In the case of compounds **1** and **2**, the correlation was not good, probably due to the difficulty in modelling the carbohydrate fragment, which is structurally very flexible. It should be noted that entropy had to be included in the calculations despite the increased computational cost. Consequently, the entropic term was often neglected. However, from preliminary tests that were performed (data not shown) it was found that the relative order of stabilities between the possible binding sites was altered when only the enthalpic term was considered.

**Figure 3.**
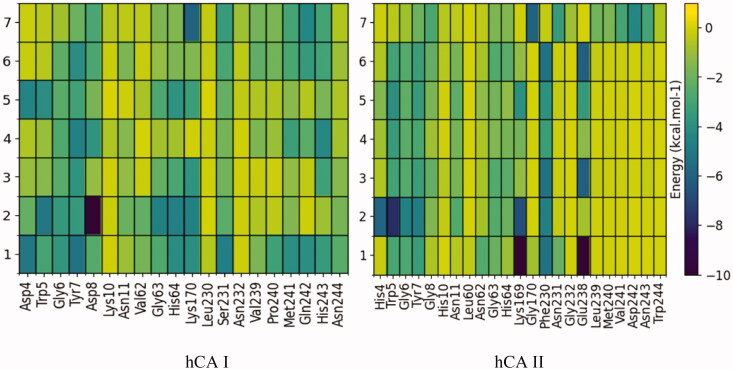
Per-residue decomposition of binding energy (only the enthalpy term considered) of ligand-bound hCA I and II, located in the pocket adjacent to the catalytic site. These calculations were performed with the MMPBSA.py module and plotted using Python's Matplotlib library. The residues shown were those that evidenced at least one interaction of < 0.3 kcal mol^−1^ with a ligand.

In order to analyse which residues could interact with the compounds, a per-residue decomposition of MM-GBSA binding energy was performed from molecular dynamics trajectories. Figure 3 shows this analysis for ligands located in the alternative pocket of CA I and CA II. In the case of the CA I pocket, there is a pronounced interaction with the residues Tyr7, Asp8, Gly63, His64, Lys170, Met241, and His243.

While the interaction in the CA II pocket occurs mainly with the residues Gly6, Tyr7, Gly63, and His64. It should be noted that **7**, in contrast to the other compounds, also interacts with the residues Leu239, Met240, Val241, Asp242, and Asn243.

**Figure 4.**
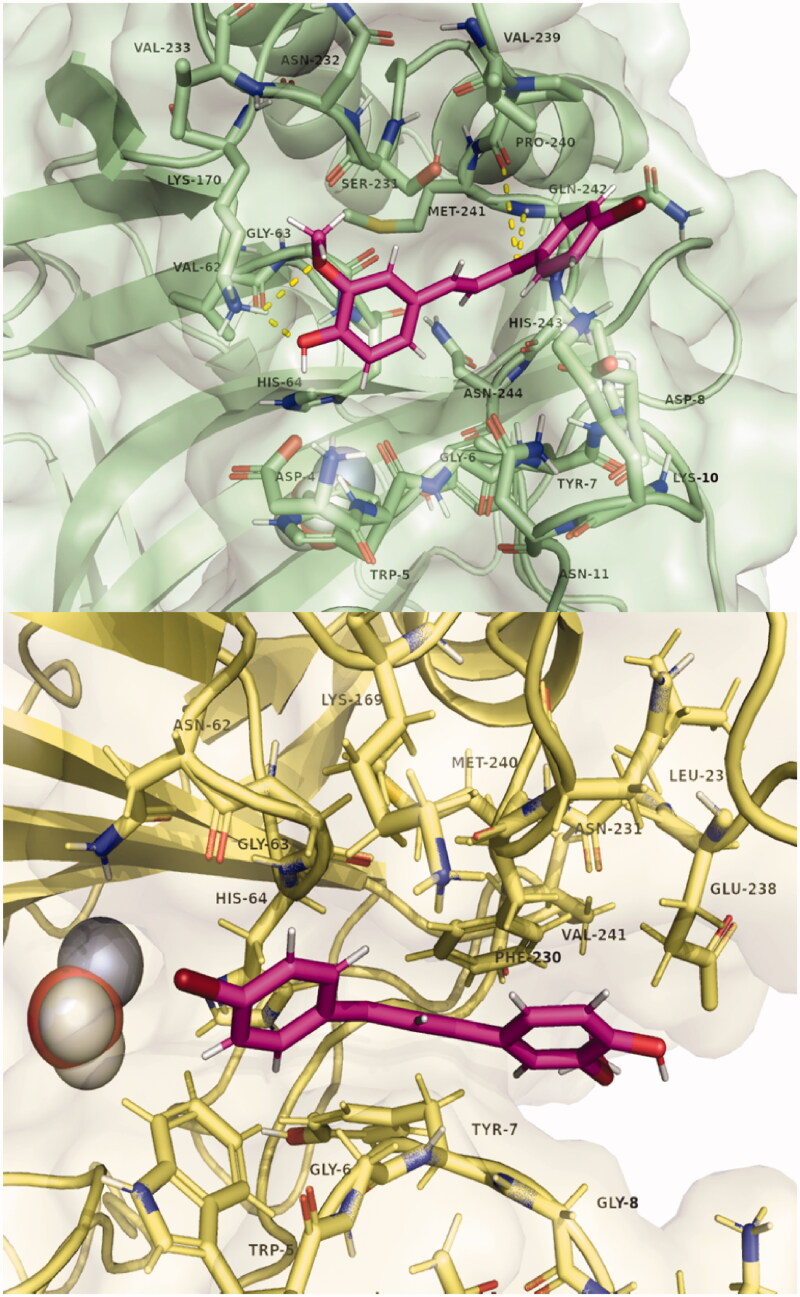
Poses of compound **7** bound in the adjacent pocket to the active site entrance of hCA I (above) and hCA II (below). Hydrogen bonds with residues Lys170, Pro 240, and Gln242 are highlighted in yellow. Images were generated with Pymol. The selected structures of all ligands with CAs are presented in the Supplementary Material.

Figure 4 shows the three-dimensional structures of compound **7** in the alternative CA I and CA II pocket. A possible explanation that we put forward initially is the possibility that this alternative pocket competes with the binding within the catalytic site. Thus, those compounds that fit within this alternative pocket do not block the catalytic site and therefore do not inhibit CA by the canonical inhibition mechanism. Along these lines, the size of this pocket is reduced in the order: CA II, CA I, CA XII, and is absent in CA IX. Precisely in this last isoform, all compounds except **7**, produced inhibition. However, computationally estimated binding energies do not support this hypothesis. From these results, the experimentally observed inhibition differences could be correlated with the interaction of the compounds with this alternative binding pocket at the entrance of the active site. Further studies, both computational and experimental, are warranted to fully reveal the mode of interaction and inhibition of these compounds with these CA isoforms.

## 4. Conclusions

In conclusion, a small series of enones **1–7** have been prepared by aldol condensation of methyl ketones, including *C*-glycosylated ones with vanillin, being obtained in moderate yields. The compounds have been investigated as inhibitors against four isozymes of CA comprising the cytosolic, ubiquitous isozymes hCA I and II as well as the transmembrane, tumour-associated isoforms hCA IX and XII which are validated antitumor targets. In this study, *C*-glycosyl and alkyl enones derivated from vanillin, have been identified as selective inhibitors of hCA IX and XII. Molecular docking studies, quantum semiempirical calculations, and molecular dynamics simulations have been carried out in order to understand the inhibition profile of the compounds **1–7** with the different CA isozymes. A good correlation was found between the calculated binding free energies and the experimental inhibitory activity. An alternative pocket, already discovered for the binding of a carboxylic acid derivative several years ago^51^, close to the active site entrance, is proposed as the site of interaction with these CAIs and could be useful in the design of potent and selective inhibitors. Further studies, both computational and experimental, are needed to fully validate the mode of interaction and inhibition of these compounds with these as well as other CA isoforms of pharmacological interest.

## Supplementary Material

Supplemental Material
