# Supplementary material for: Vanillin enones as selective inhibitors of the cancer associated carbonic anhydrase isoforms IX and XII. The out of the active site pocket for the design of selective inhibitors?
Source: J Enzyme Inhib Med Chem. 2021 Oct 4;36(1):2118–27. doi: 10.1080/14756366.2021.1982933 (PMC8510595; doi:10.1080/14756366.2021.1982933)

Supplementary information for

Vanillin enones as selective inhibitors of the cancer associated carbonic anhydrase isoforms IX and XII. The out of the active site pocket for the design of selective inhibitors ?

Leonardo E. Riafrecha[a], Macarena S. Le Pors[a], Martín J. Lavecchia[b], Silvia Bua[c], Claudiu T. Supuran[c,d,*] and Pedro A. Colinas[a,*]

[a]CEDECOR (UNLP-CICBA), CONICET, Departamento de Química, Facultad de Ciencias Exactas, Universidad Nacional de La Plata, 47 y 115, 1900 La Plata, Argentina
[b]CEQUINOR (CONICET-UNLP) Facultad de Ciencias Exactas, Universidad Nacional de La Plata, 47 y 115, 1900 La Plata, Argentina
[c]Universitá degli Studi di Firenze, Laboratorio di Chimica Bioinorganica, Rm. 188, Via della Lastruccia 3, I-50019 Sesto Fiorentino (Florence), Italy
[d]Università degli Studi di Firenze, NEUROFARBA Department, Section of Pharmaceutical Chemistry, Via Ugo Schiff 6, 50019 Sesto Fiorentino (Florence), Italy

Index

1. Chemistry..S2
1.1. Molecular Docking S2-S21
FigureS1..S2
TableS1..S3
FigureS2..S4-S13
FigureS3..S14-S21
2. Experimental..S22
2.1 NMR snapshots.. …S22-S28
1-(β-D-glucopiranosyl)-4-(4-hydroxy-3-methoxyphenyl)but-3-en-2-one (1)..S22
1-(β-D-galactopiranosyl)-4-(4-hydroxy-3-methoxyphenyl)but-3-en-2-one (2)..S23
(*E*)-4-(4-hydroxy-3-methoxyphenyl)but-3-en-2-one (3)..S24
(*E*)-1-(4-hydroxy-3-methoxyphenyl)pent-1-en-3-one (4)..S25
(*E*)-1-(4-hydroxy-3-methoxyphenyl)-5-methylhex-1-en-3-one (5)..S26
(*E*)-3-(4-hydroxy-3-methoxyphenyl)-1-phenylprop-2-en-1-one (6)..S27
(*E*)-1-(4-bromophenyl)-3-(4-hydroxy-3-methoxyphenyl)prop-2-en-1-one (7)..S28

1. Chemistry

1.1. Molecular Docking

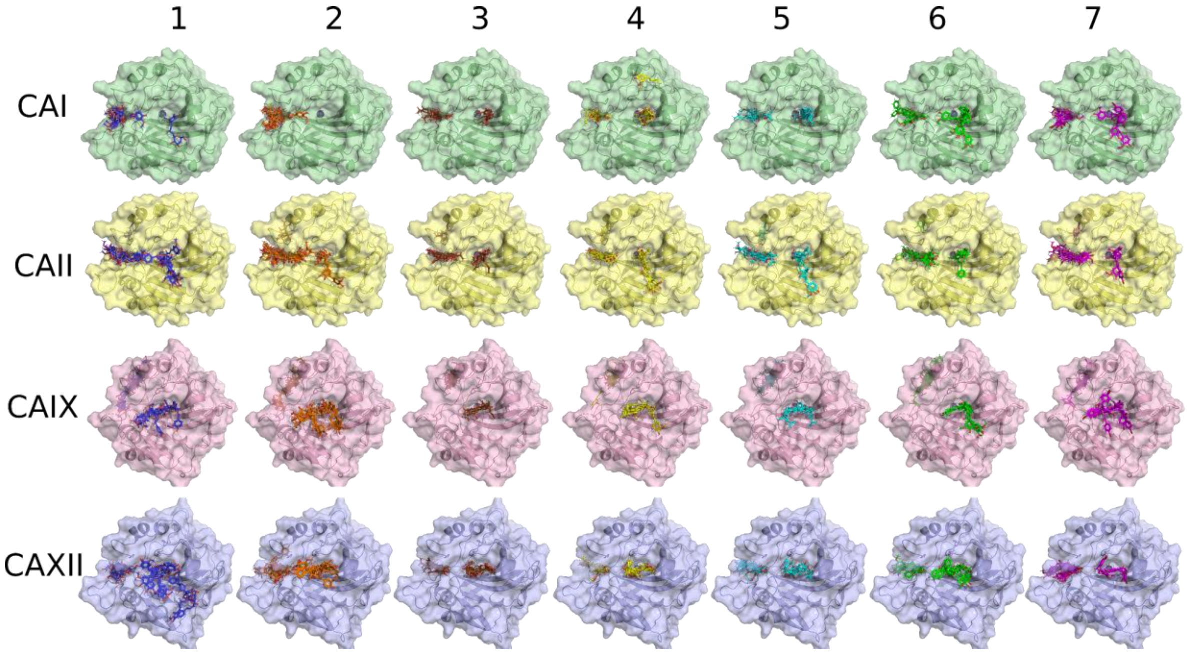

Figure S1. Ligand poses obtained with docking on the carbonic anhydrase isozymes.

Table S1. Binding enthalpies (kcal/mol) calculated with PM6 and MOZYME approach using MOPAC. Location classification of ligand poses: *down*, poses placed inside the catalytic cavity with the methoxyphenol close the Zn^{2+} cation (distance <3Å); *up*, poses localized inside the catalytic cavity and close de Zn^{2+} cation (distance <3Å) with outward-facing methoxyphenol; *top down* and *top up*, poses that also block the catalytic site at a distance between 3Å and 7Å from the Zn^{2+} cation; *pocket*, poses located in the adjacent alternative pocket (in case of CAIX, poses at a distance < 3.5Å from pocket residues); *unclassified*, poses that were not located at the catalytic site or in the neighboring pocket.

Compound	Location	CAI (2FW4)	CAII (3KS3)	CAIX (6FE2)	CAXII (1JCZ)
**1**	down	-	-	-	-
	up	-	-	-	-
	top down	-17	-12	-33	-28
	top up	-	-24	-30	-22
	pocket	-34	-28	-26	-33
	unclassified	-1	-27	-24	-32
**2**	down	-	-	-22	-
	up	-	-	-	-
	top down	-	-	-20	-26
	top up	-	-25	-34	-18
	pocket	-43	-28	-28	-42
	unclassified	-	-18	-33	-32
**3**	down	-	-	-	-
	up	-	-	9	-
	top down	-6	0	-17	-7
	top up	5	-17	-6	-1
	pocket	-17	-15	-	-24
	unclassified	-1	-11	-18	-
**4**	down	-	-	-	-
	up	10	-	2	-
	top down	-9	-7	-17	-8
	top up	3	-	-9	-7
	pocket	-16	-19	-	-23
	unclassified	-15	-18	-20	-
**5**	down	-	-	-9	-
	up	2	-	-	-
	top down	-10	-9	-18	-10
	top up	6	-	-13	-20
	pocket	-20	-17	-	-28
	unclassified	-	-13	-21	-
**6**	down	-	-	-	-
	up	-	-	-10	-
	top down	-15	-5	-21	-21
	top up	-22	-5	-20	-4
	pocket	-20	-21	-18	-37
	unclassified	-	-16	-26	-
**7**	down	-	-	-	-
	up	-	-	-	-
	top down	-18	-15	-26	-14
	top up	0	-10	-	-3
	pocket	-18	-22	-24	-27
	unclassified	-24	-16	-26	-

Figure S2. RMSD profiles of the equilibration/production stage are shown for CA I with ligands. “RMSD Protein” was measured considering all residues in the complex aligned using the protein backbone. The plots were generated with CPPTRAJ and GNUPLOT.

**Ligand**	**Location**	**RMSD**
**1**	catalytic down	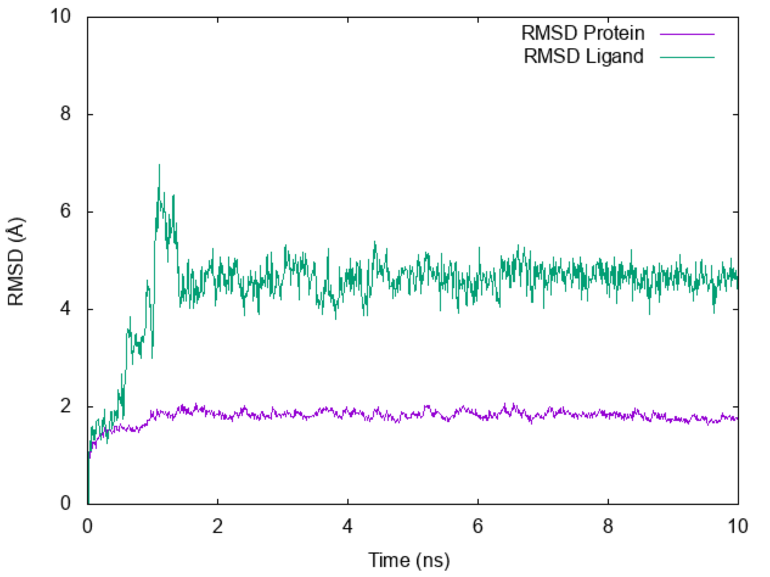
	pocket	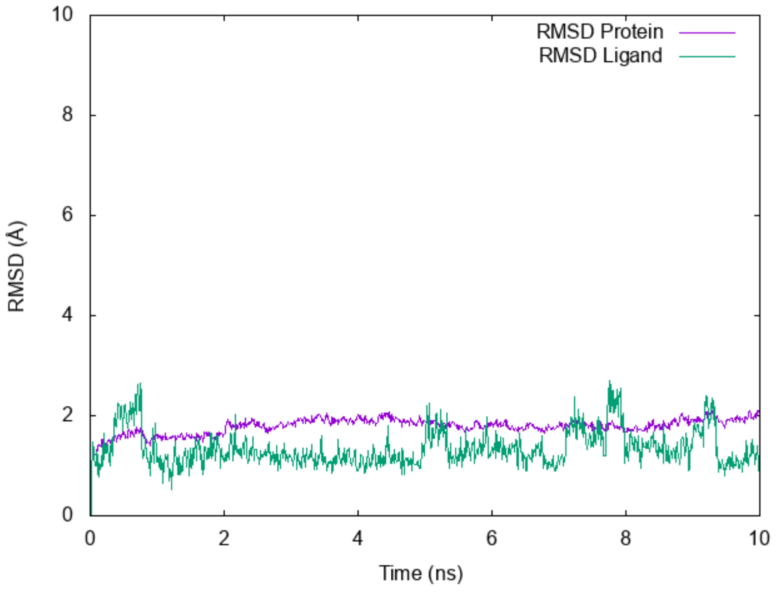

**2**	catalytic down	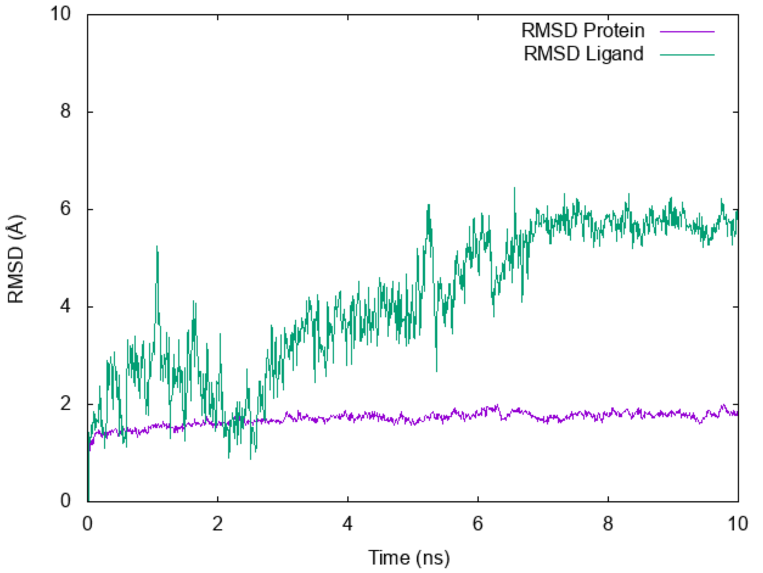
	pocket	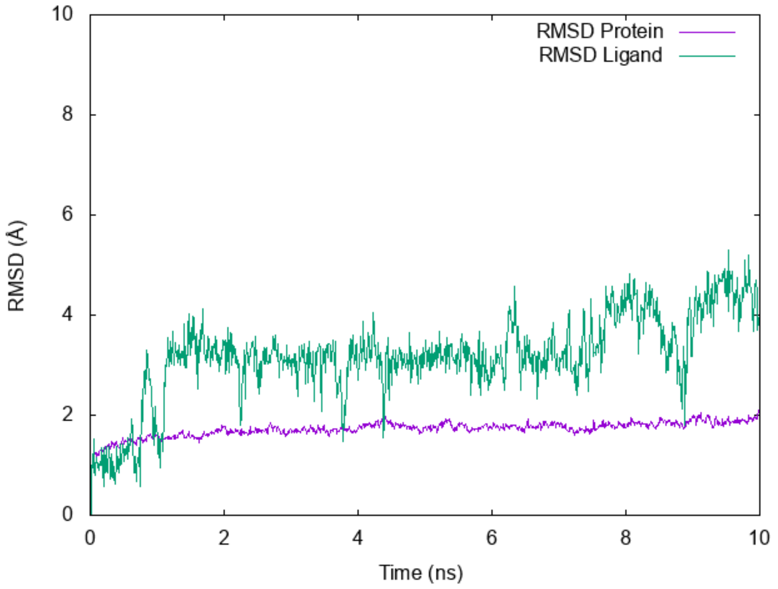

<table>
<tr><td rowspan="2">3</td><td>catalytic down</td><td>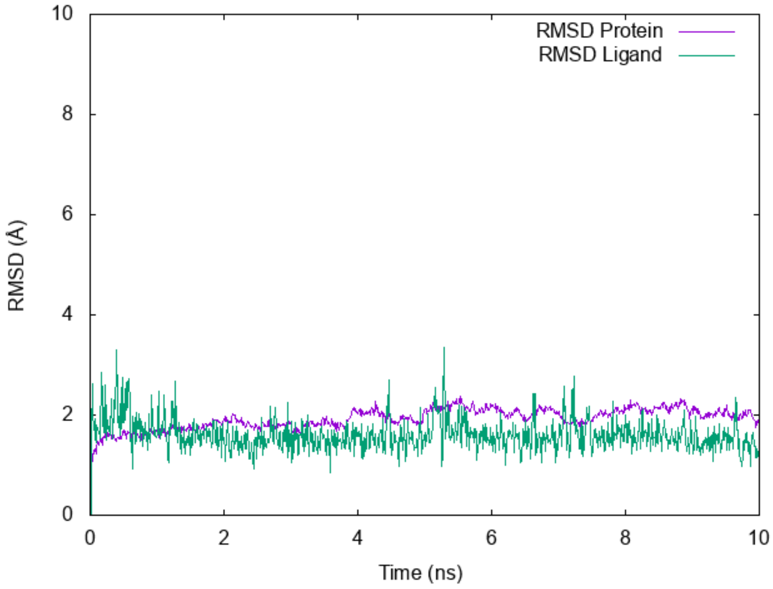
</td></tr>
<tr><td>catalytic up</td><td>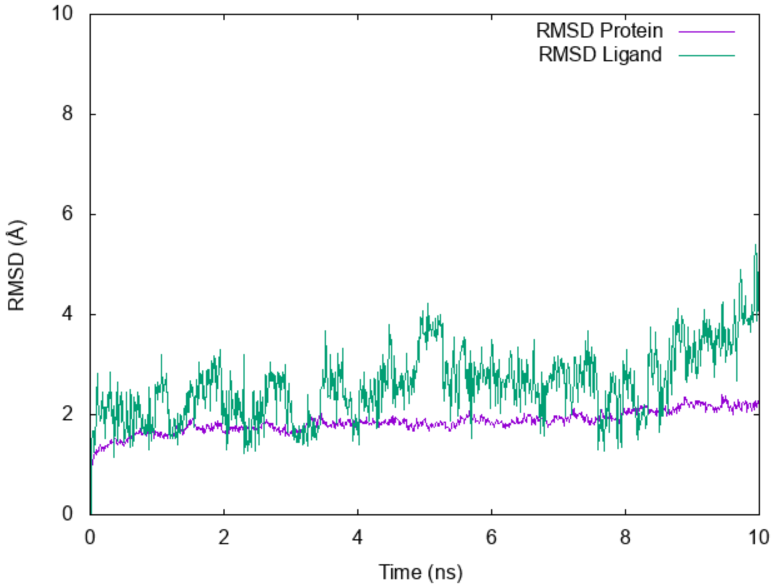
</td></tr>
</table>

	pocket	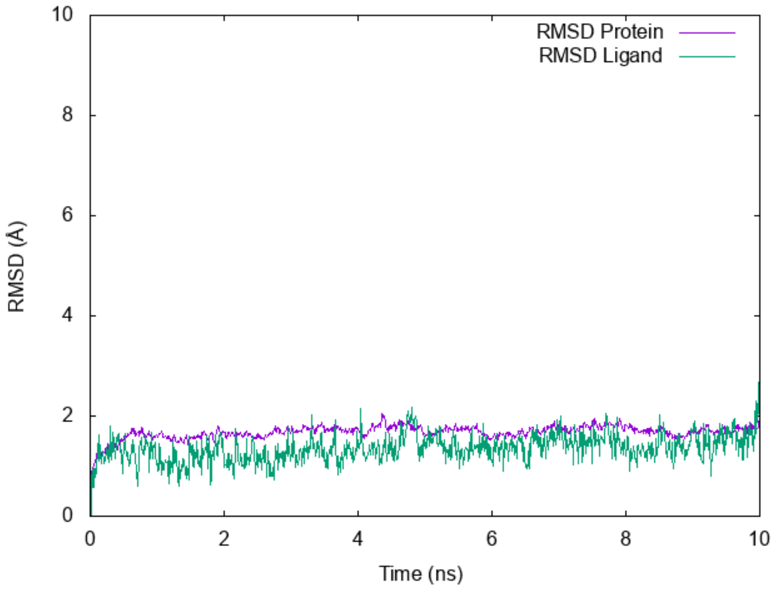
**4**	catalytic down	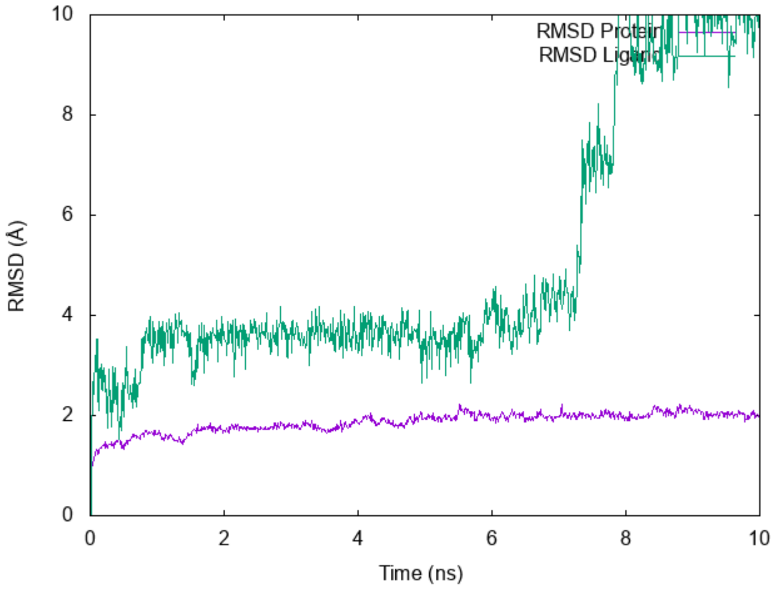

	catalytic up	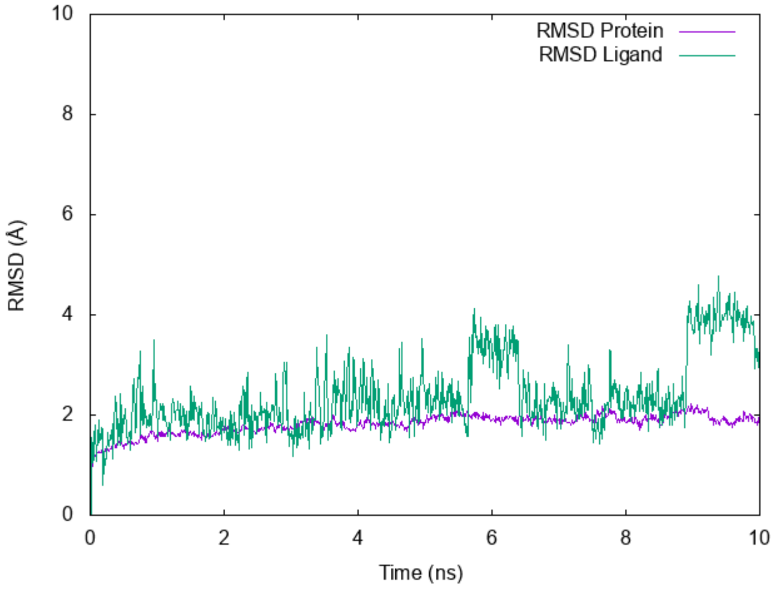
	pocket	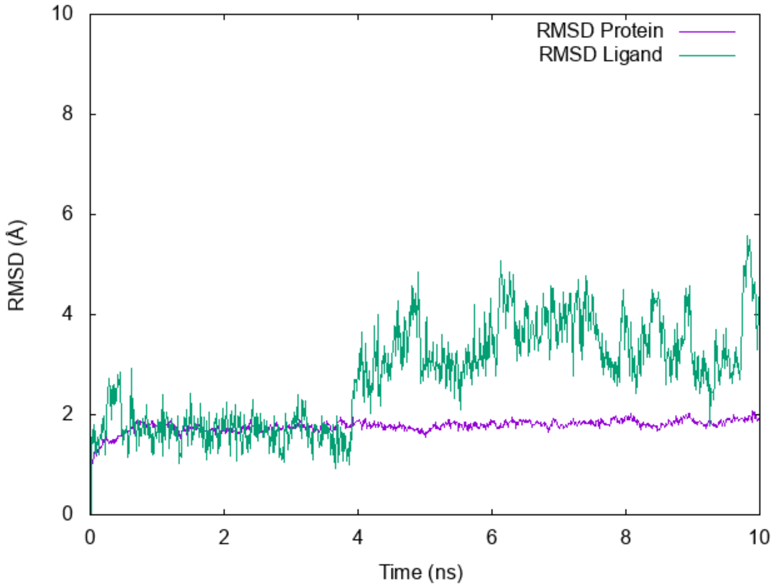

**5**	catalytic down	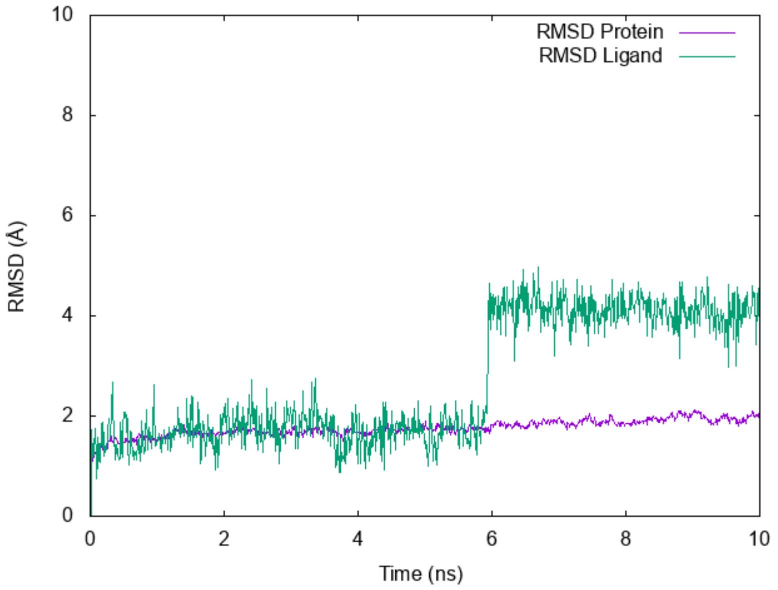
	catalytic up	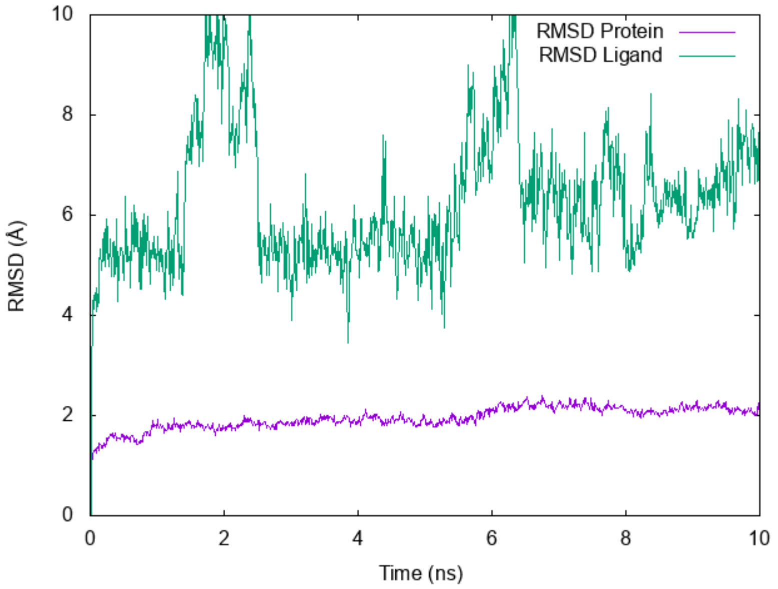

<table>
<tr><td></td><td>pocket</td><td>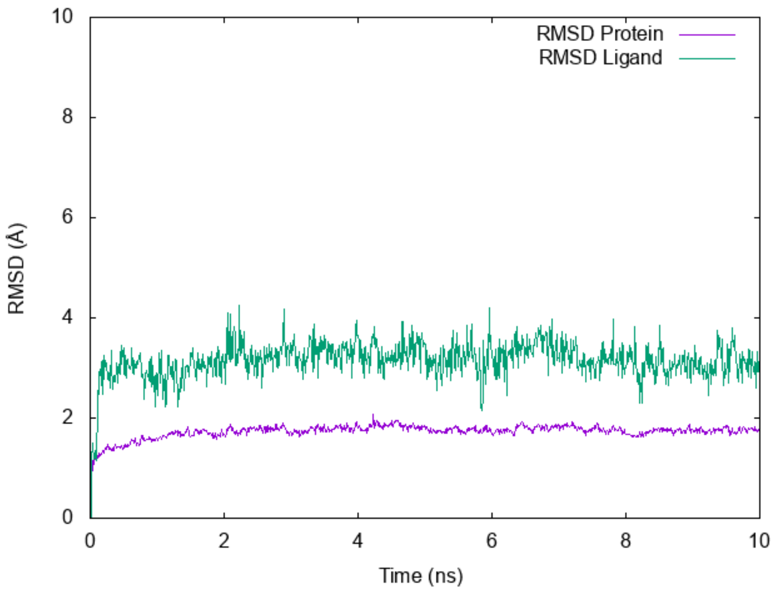
</td></tr>
<tr><td>6</td><td>catalytic
down</td><td>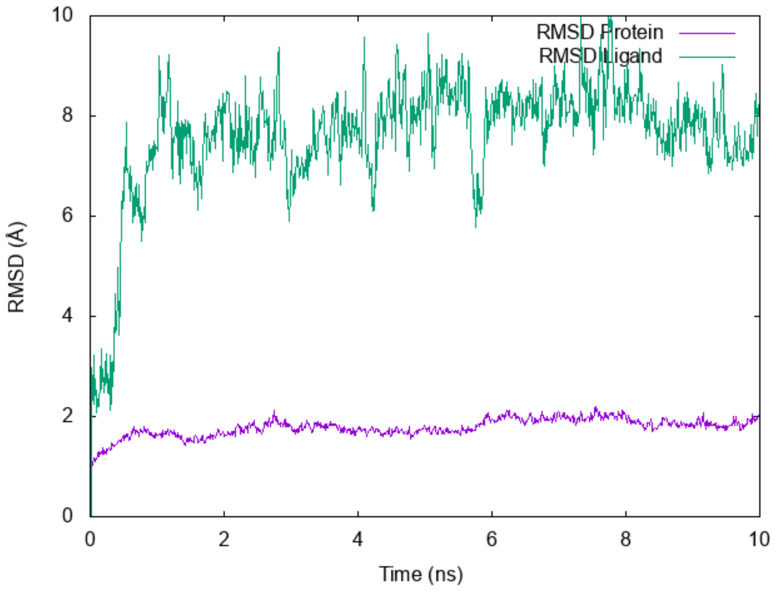
</td></tr>
</table>

<table>
<tr><td></td><td>catalytic up</td><td>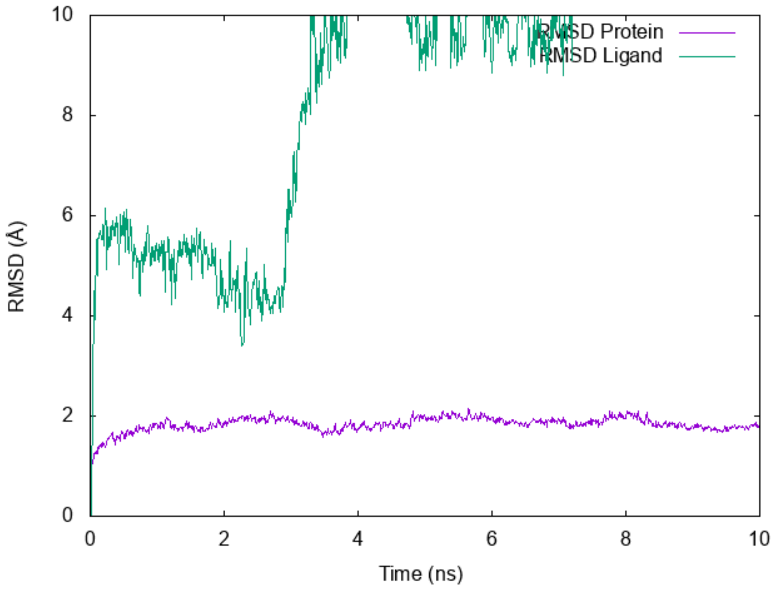
</td></tr>
<tr><td></td><td>pocket</td><td>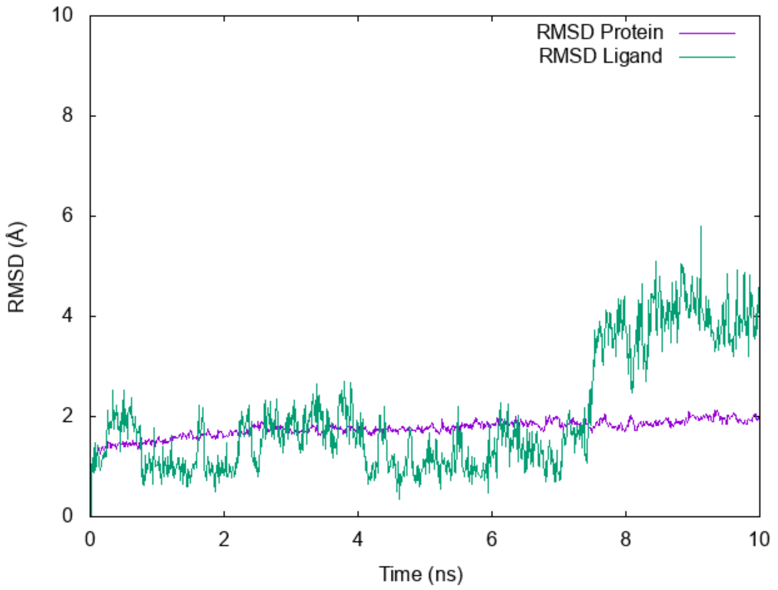
</td></tr>
</table>

**7**	catalytic down	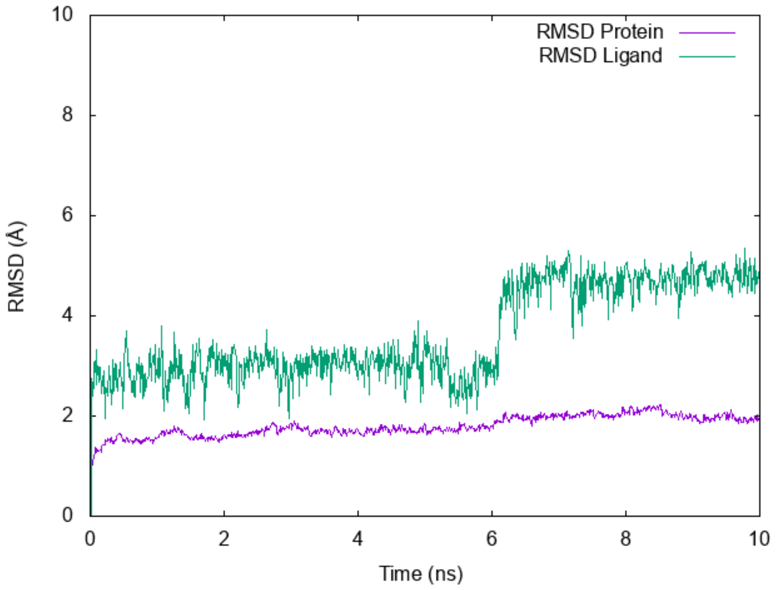
	catalytic up	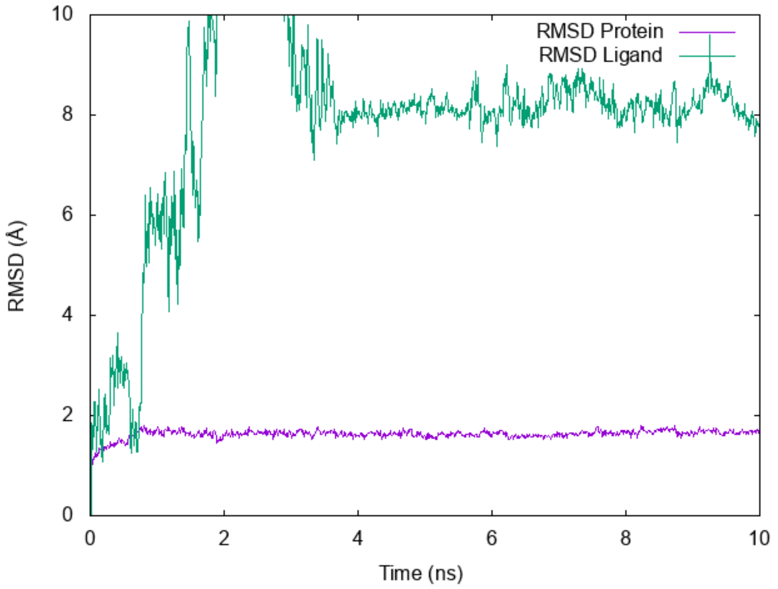

	pocket	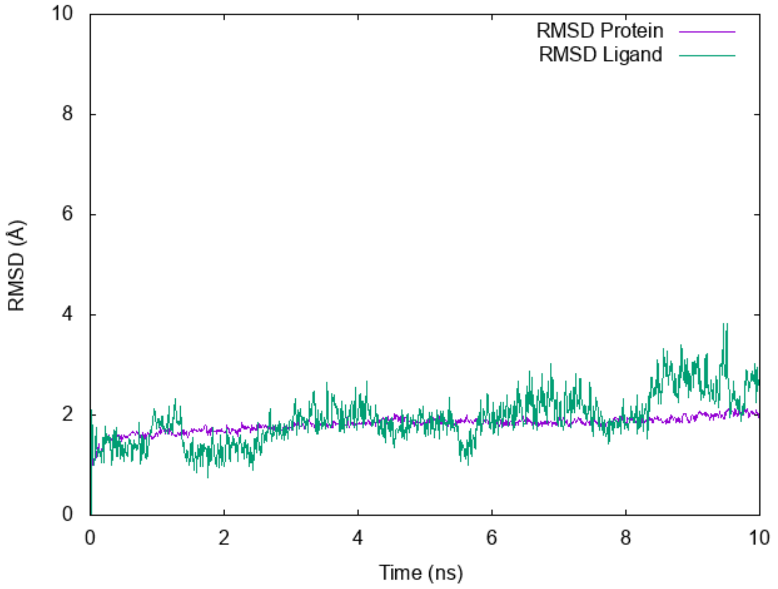

Figure S3. RMSD profiles of the equilibration/production stage are shown for CA II with ligands. "RMSD Protein" was measured considering all residues in the complex aligned using the protein backbone. The plots were generated with CPPTRAJ and GNUPLOT.

Ligand	Location	RMSD
**1**	catalytic down	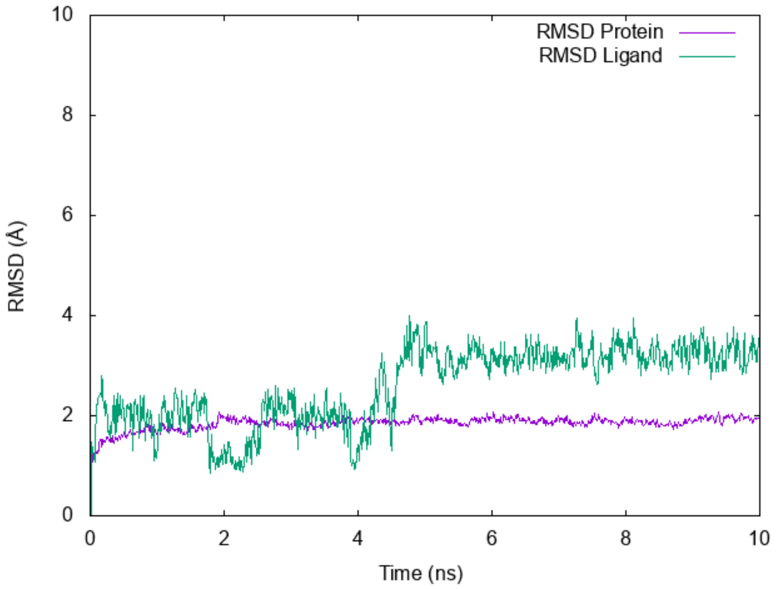
	pocket	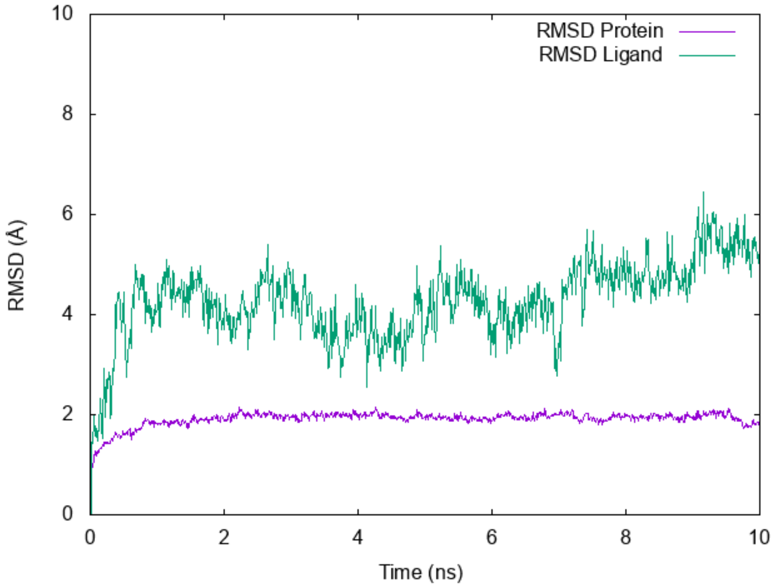

**2**	catalytic down	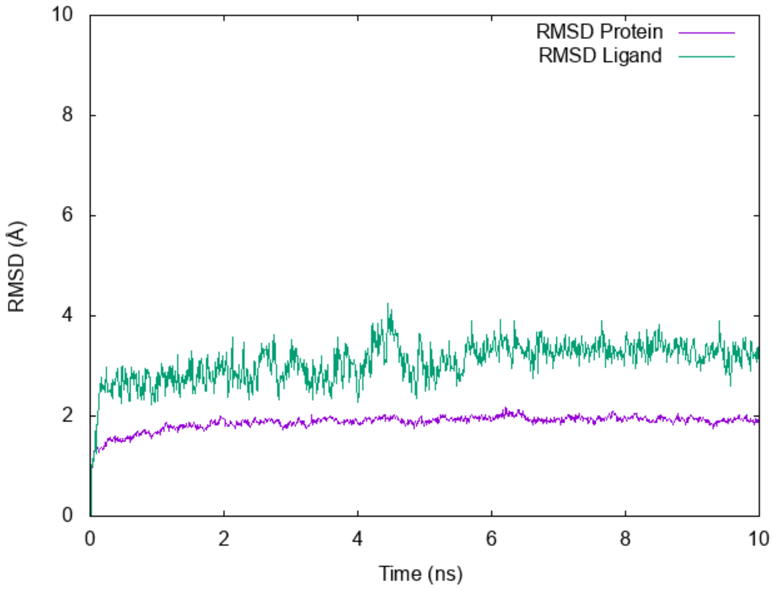
	pocket	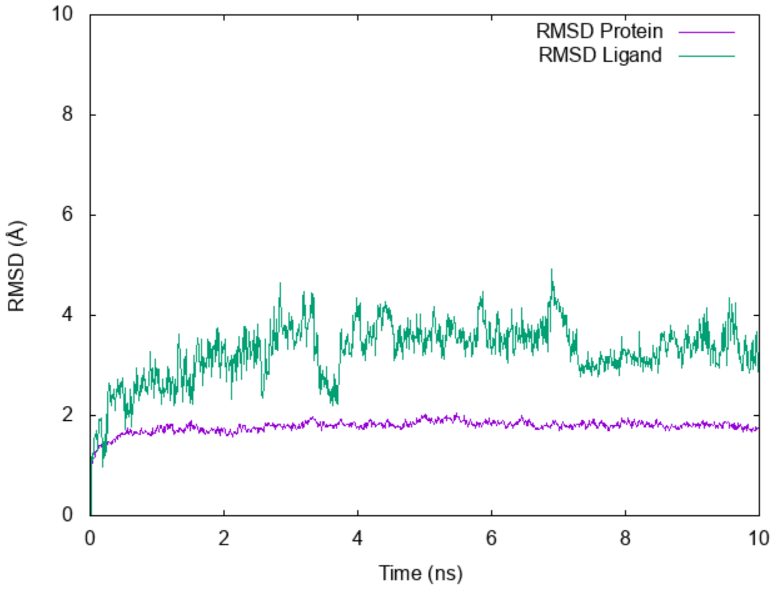

**3**	catalytic down	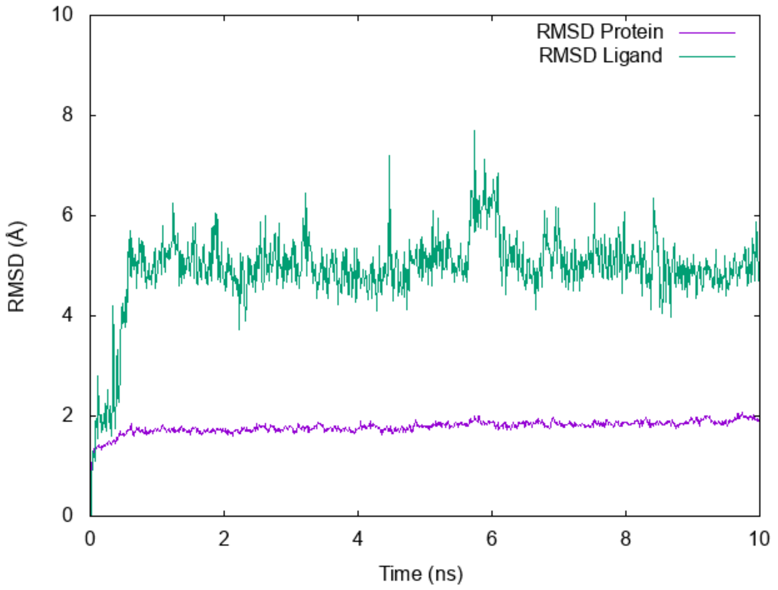
	catalytic up	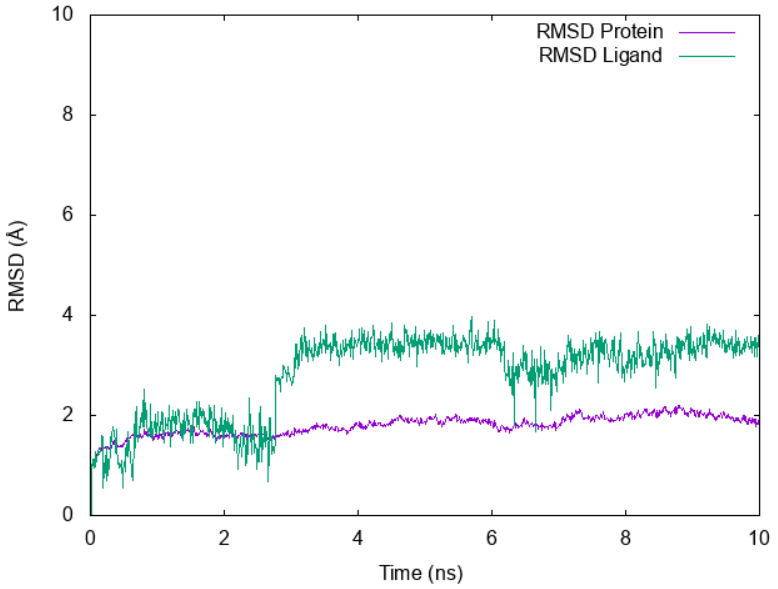

	pocket	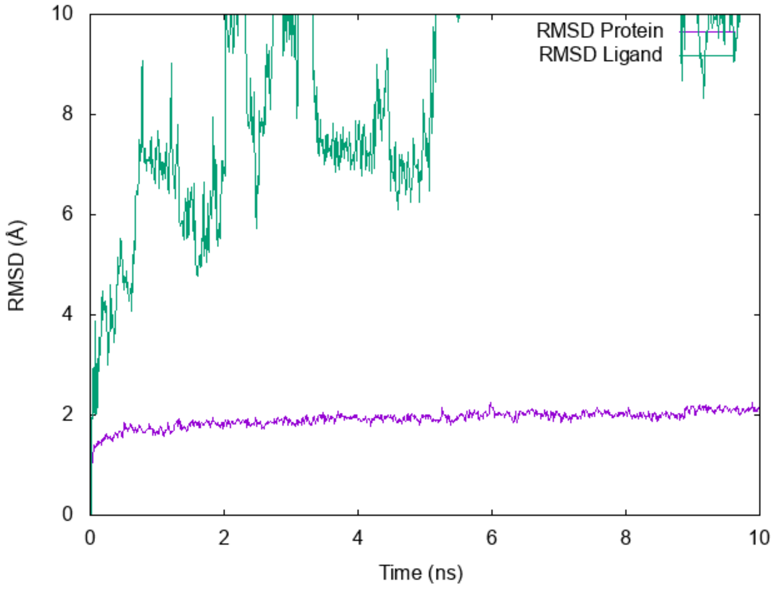
**4**	catalytic down	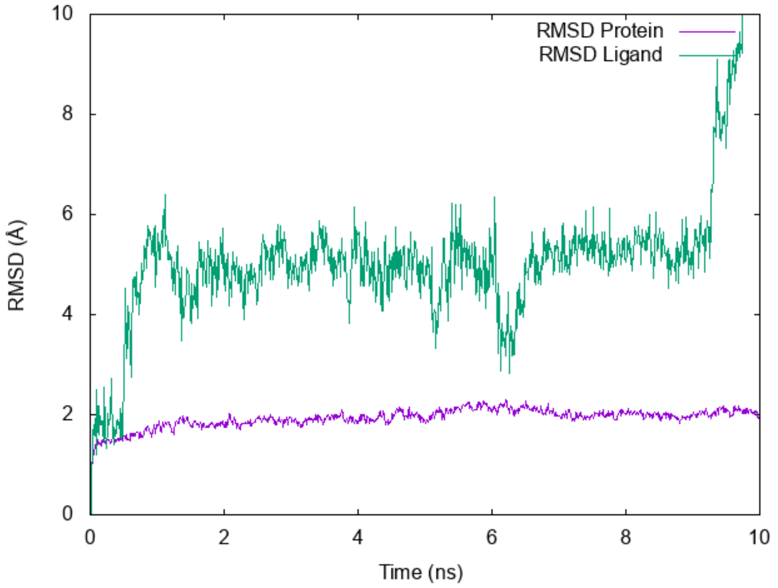

	catalytic up	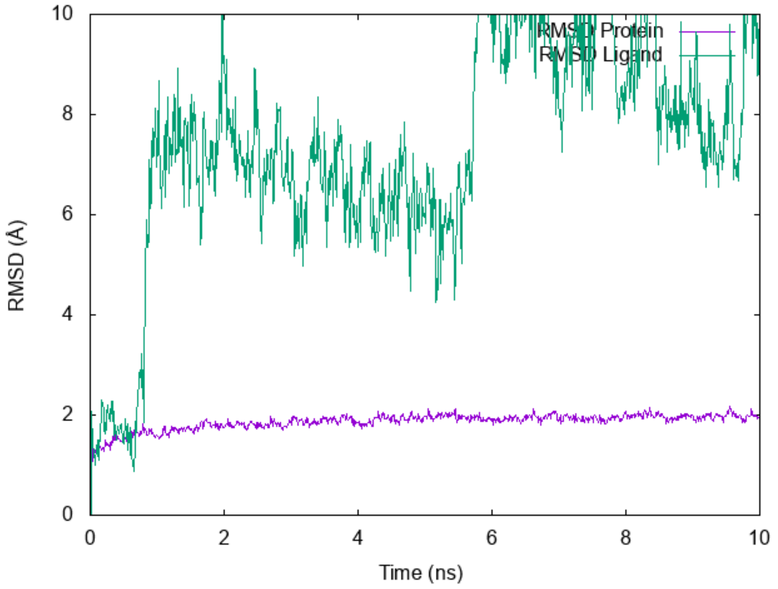
	pocket	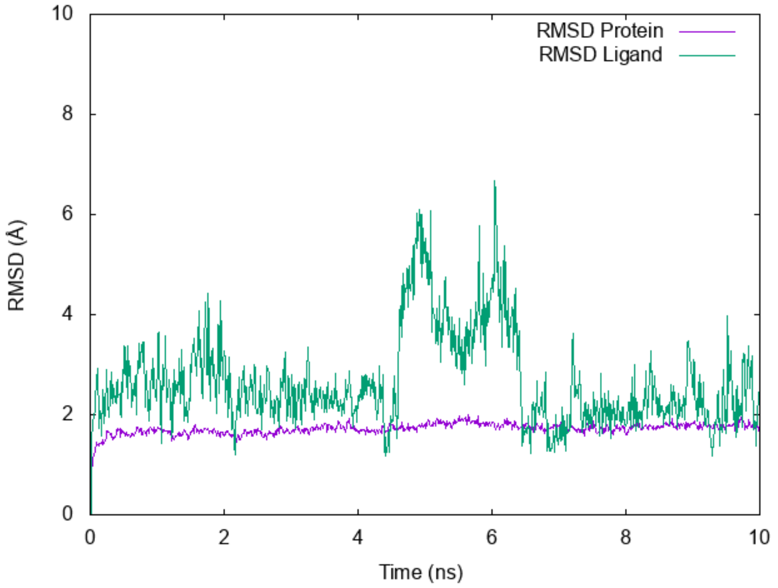

**5**	catalytic down	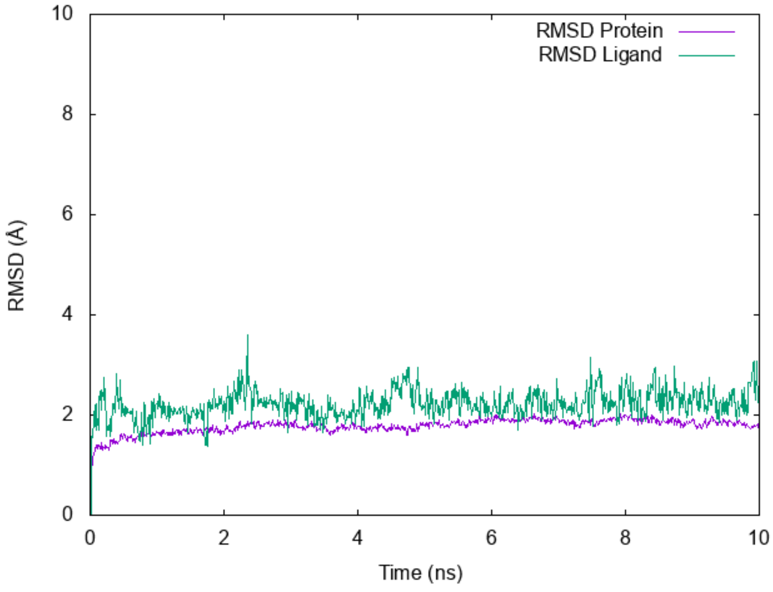
	pocket	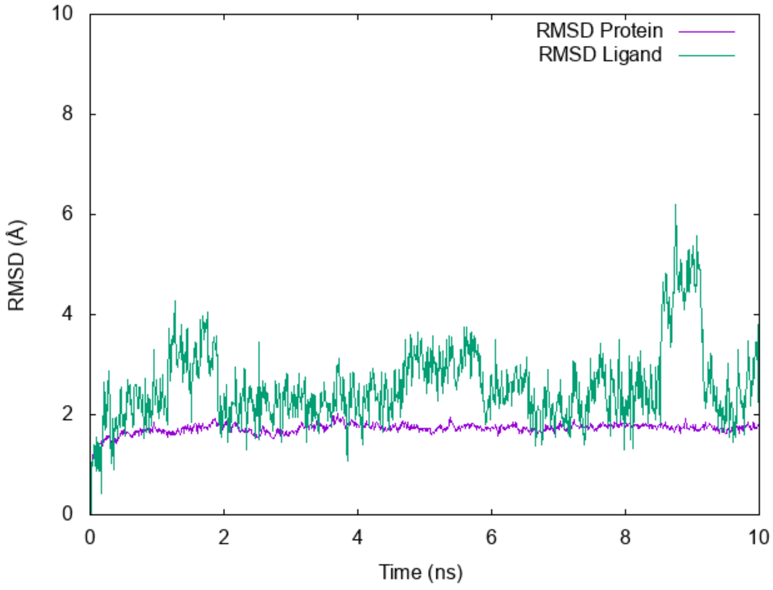

**6**	catalytic down	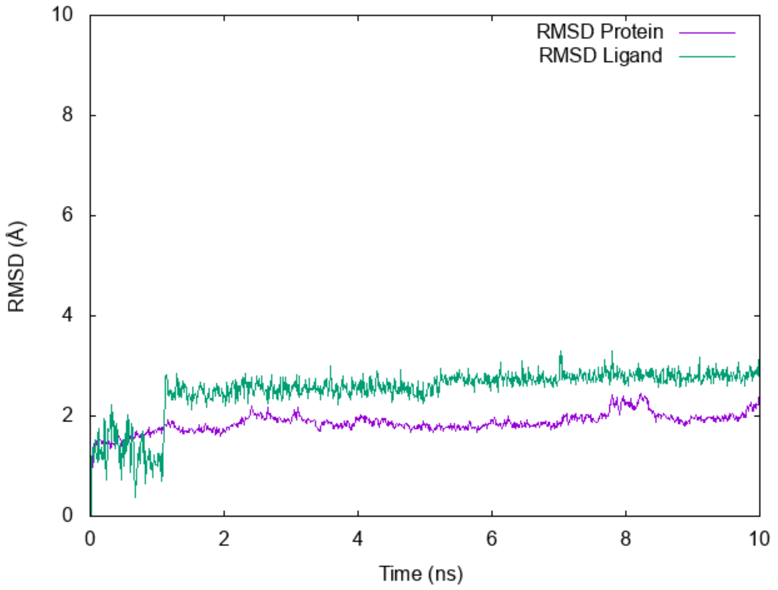
	pocket	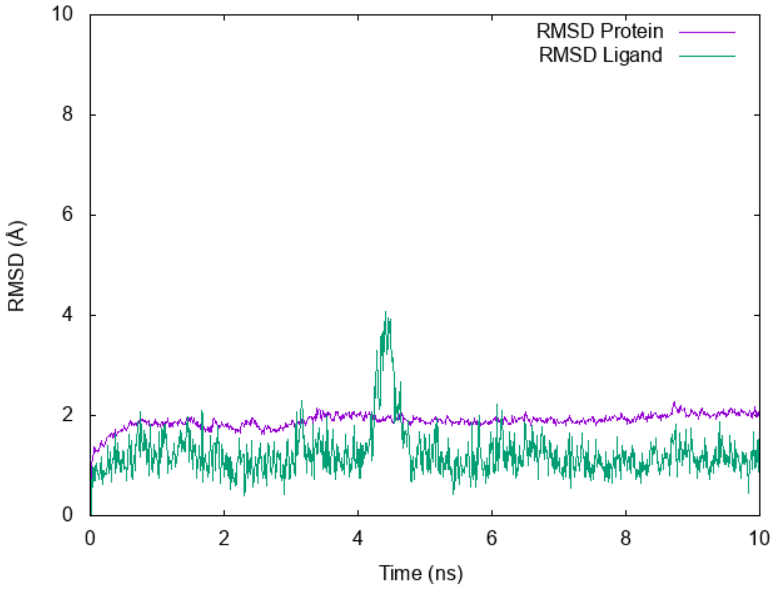

**7**	catalytic down	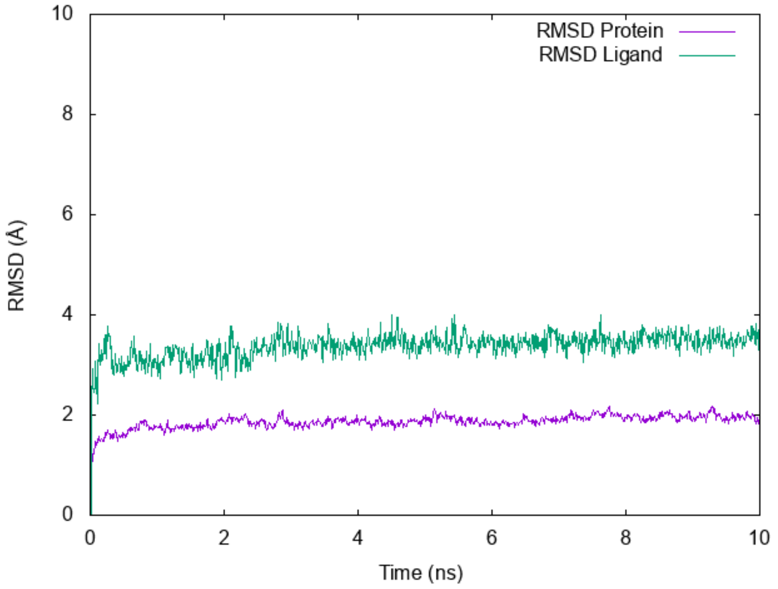
	pocket	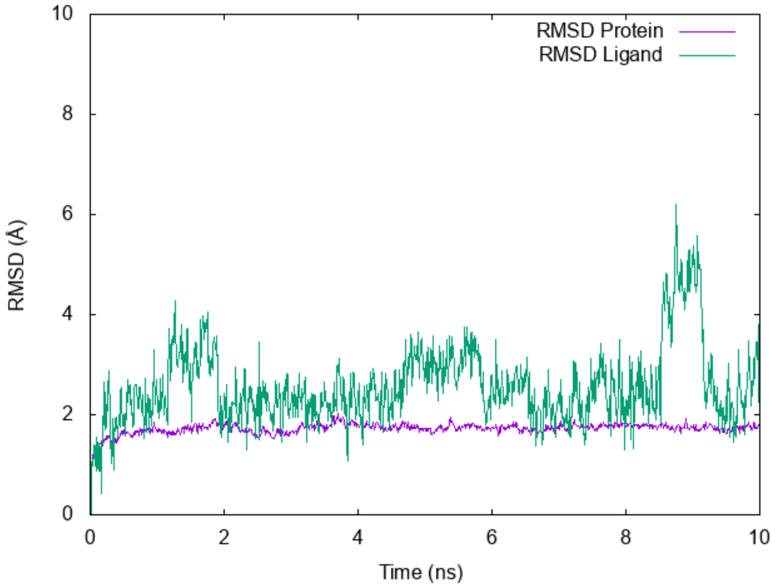

2. Experimental

2.1 NMR snapshots

1-(β-D-glucopiranosyl)-4-(4-hydroxy-3-methoxyphenyl)but-3-en-2-one (1)

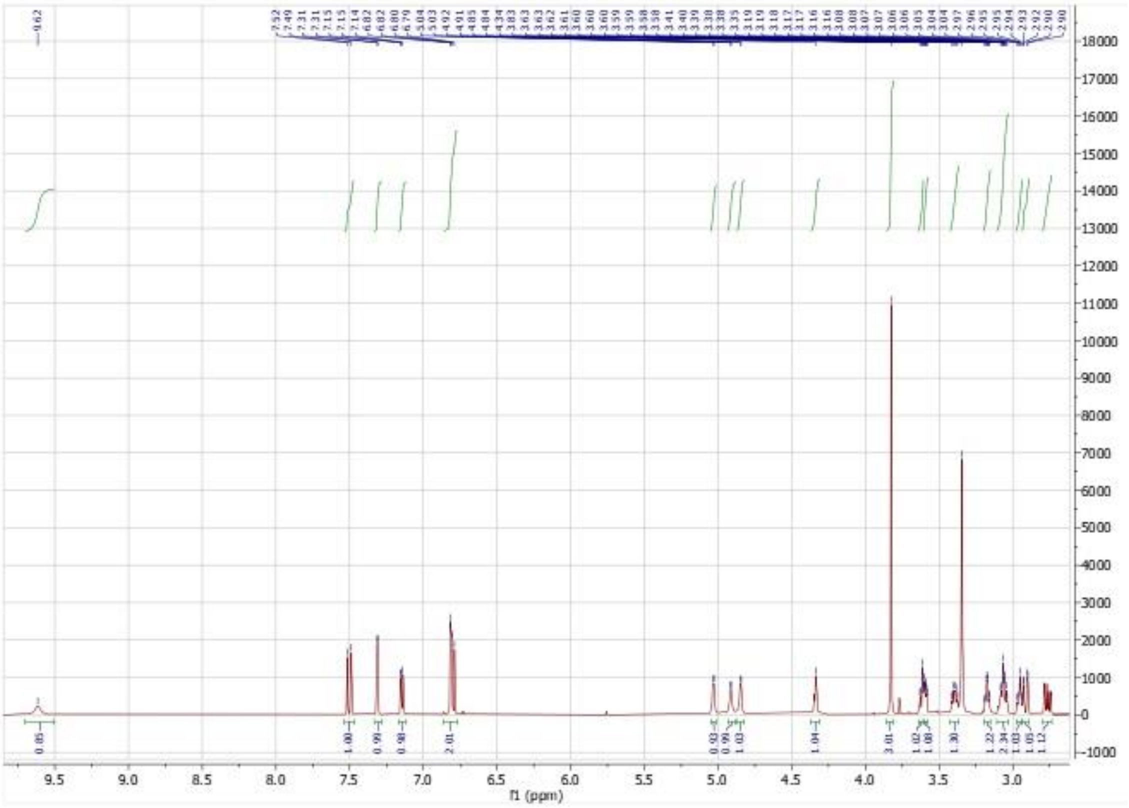

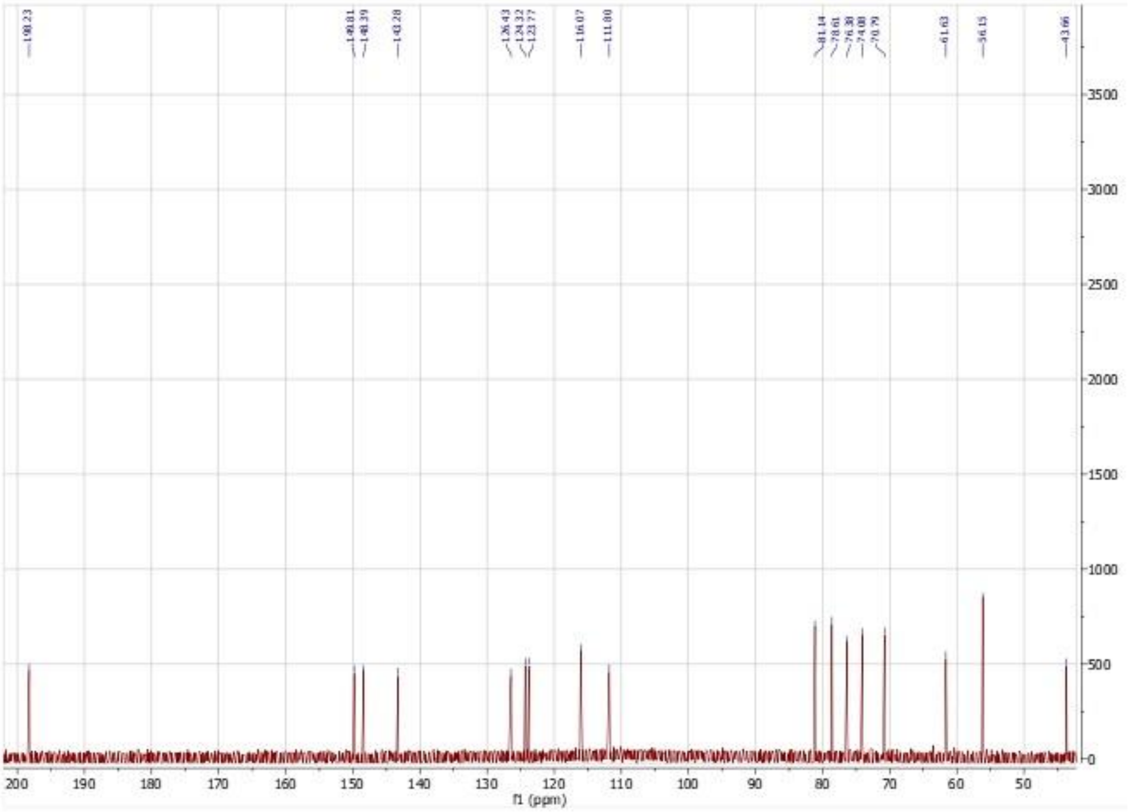

1-(β-D-galactopiranosyl)-4-(4-hydroxy-3-methoxyphenyl)but-3-en-2-one (2)

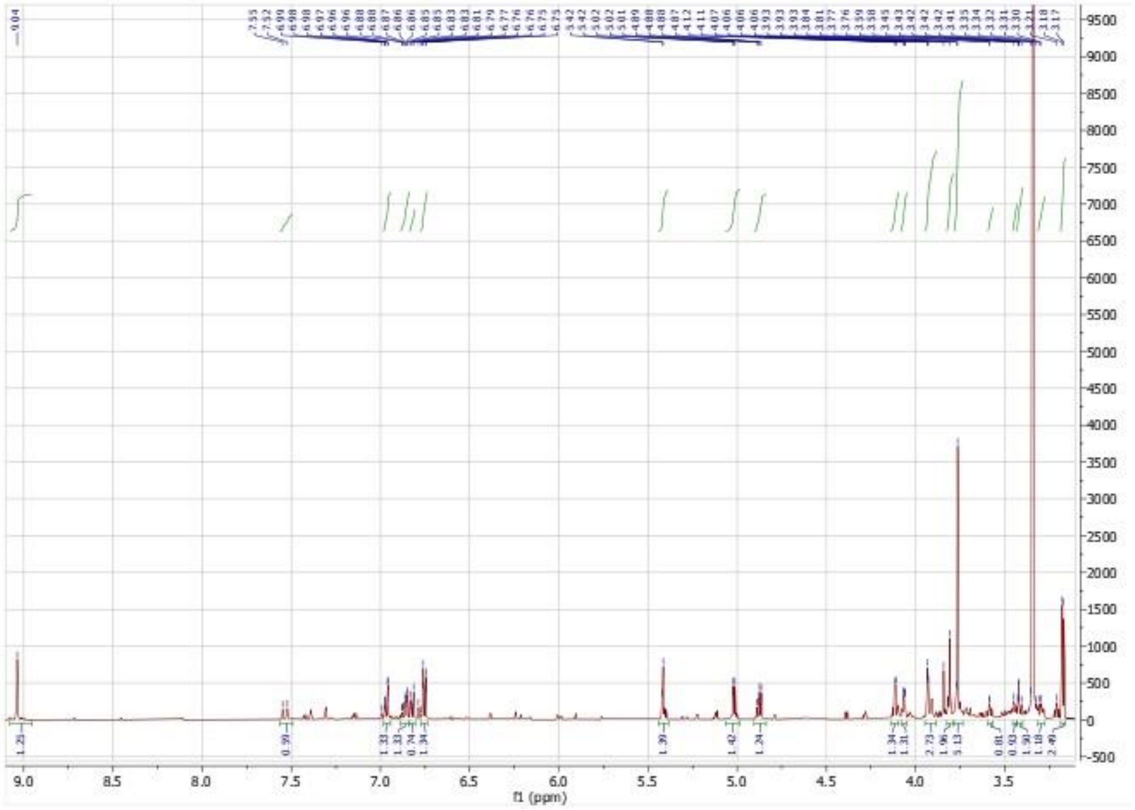

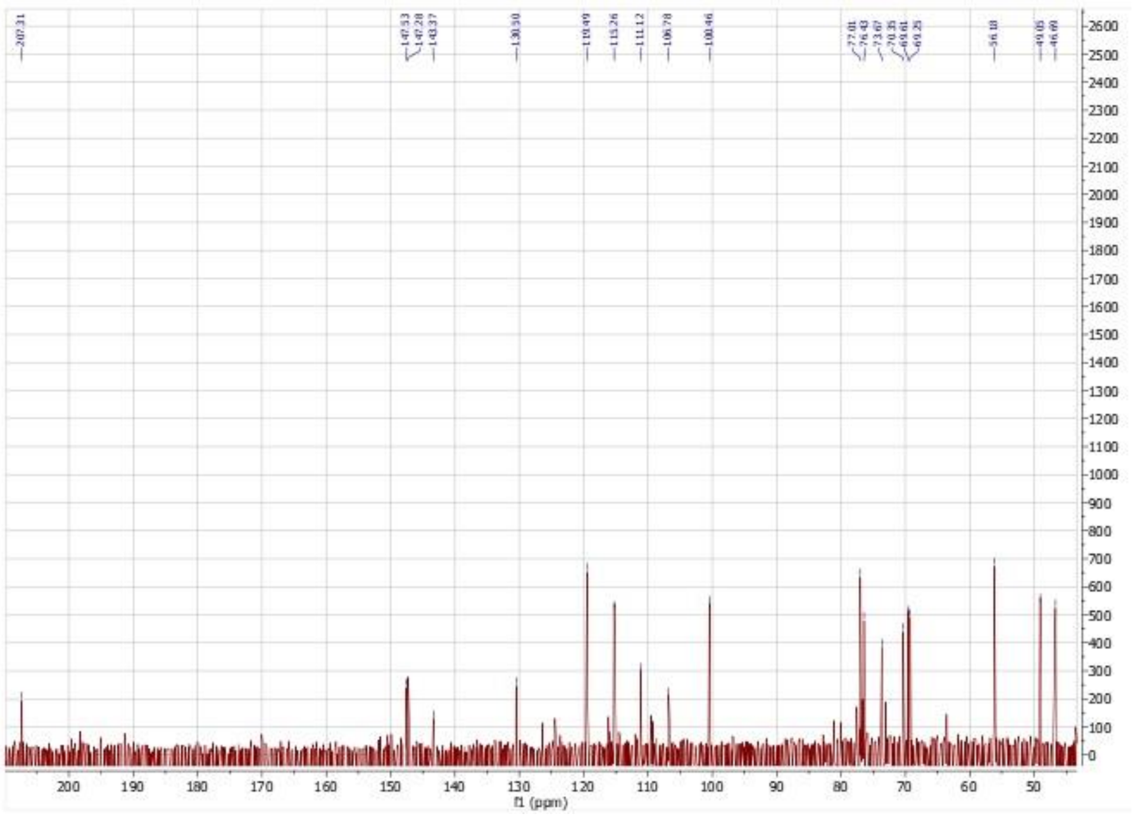

(*E*)-4-(4-hydroxy-3-methoxyphenyl)but-3-en-2-one (3)

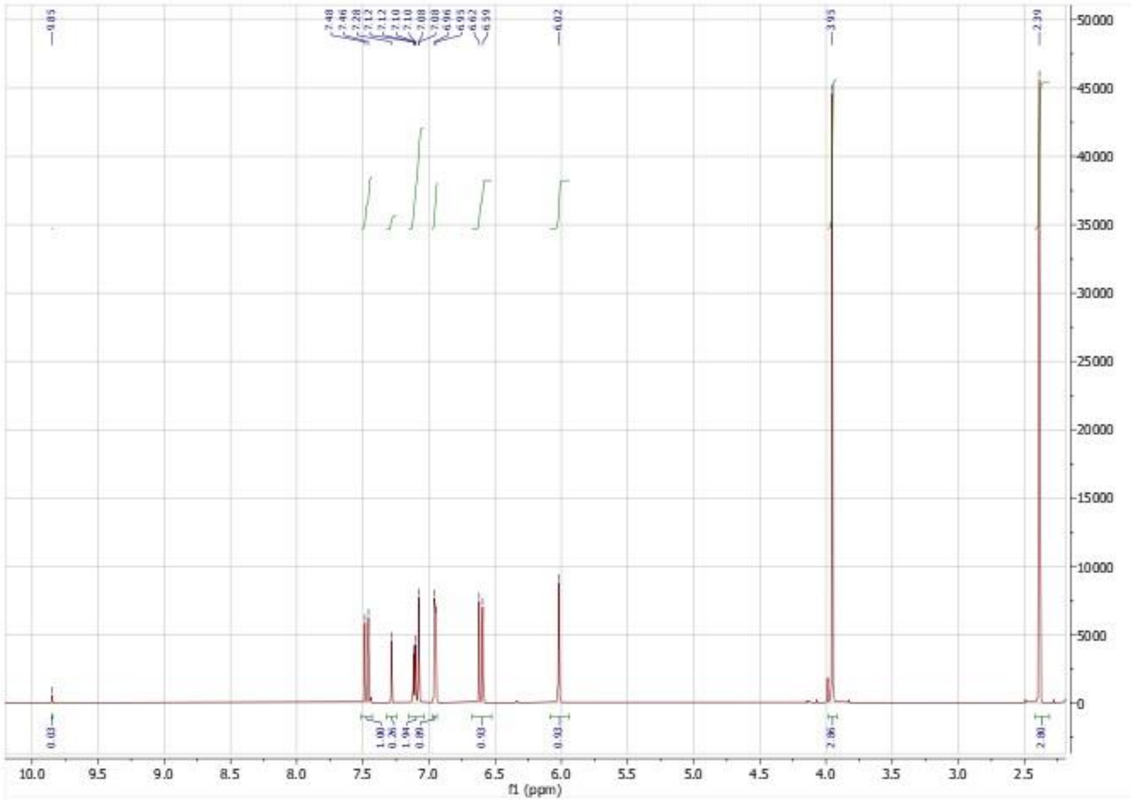

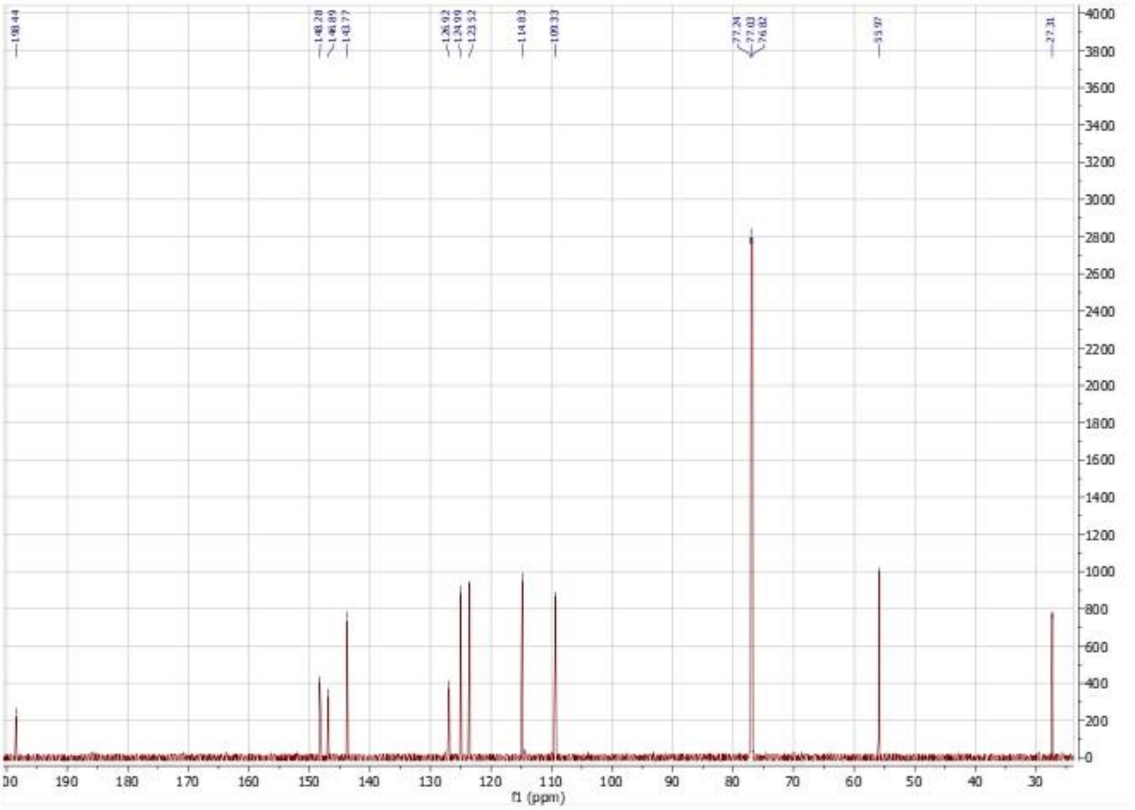

(*E*)-1-(4-hydroxy-3-methoxyphenyl)pent-1-en-3-one (4)

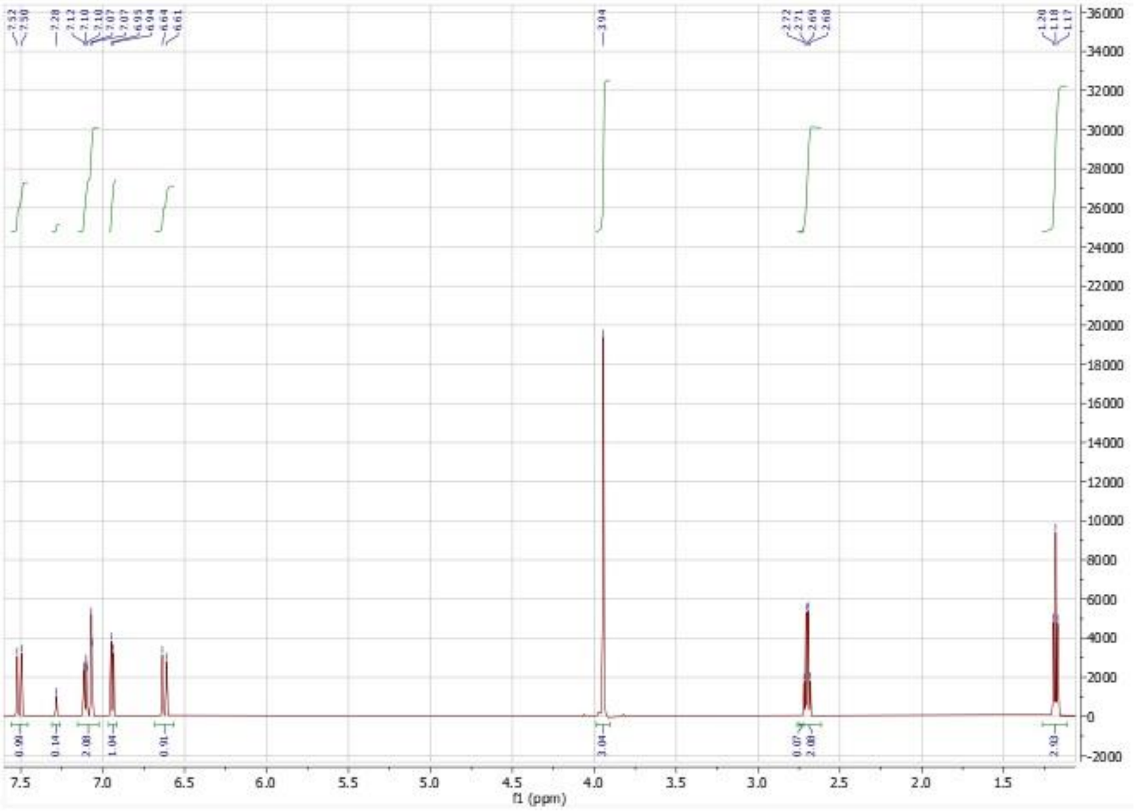

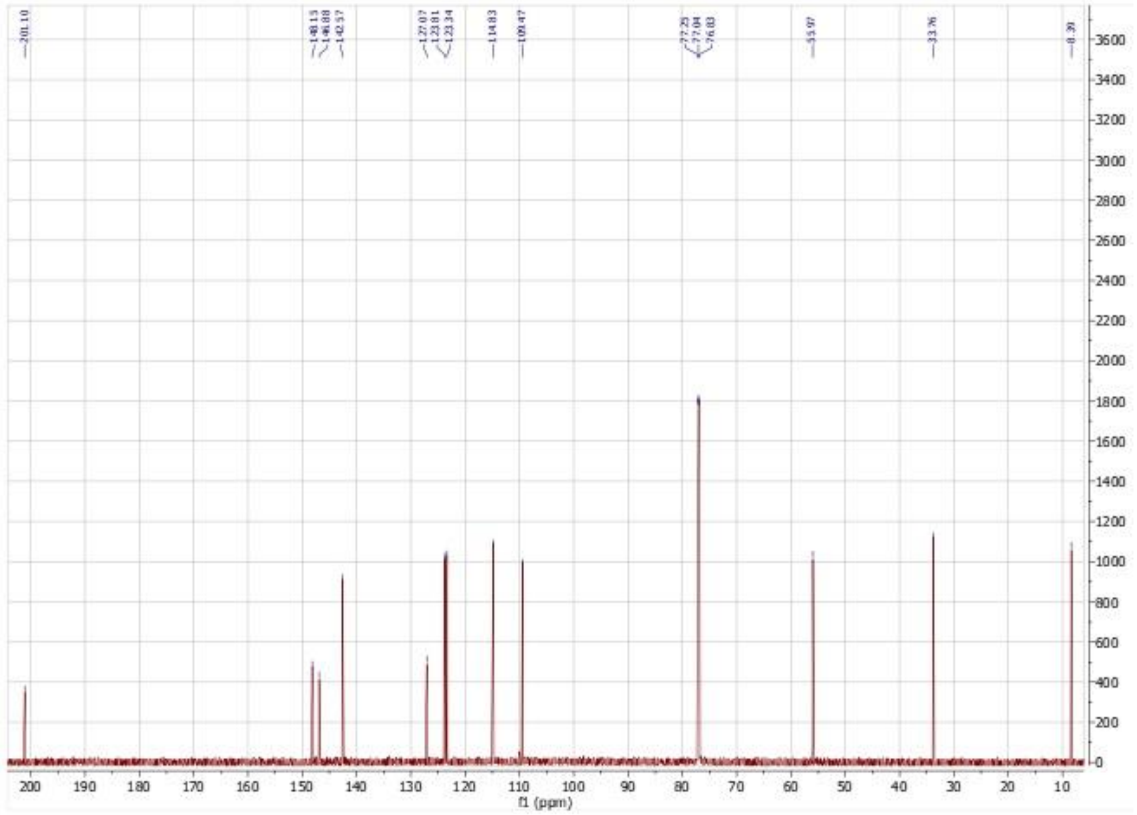

(*E*)-1-(4-hydroxy-3-methoxyphenyl)-5-methylhex-1-en-3-one (5)

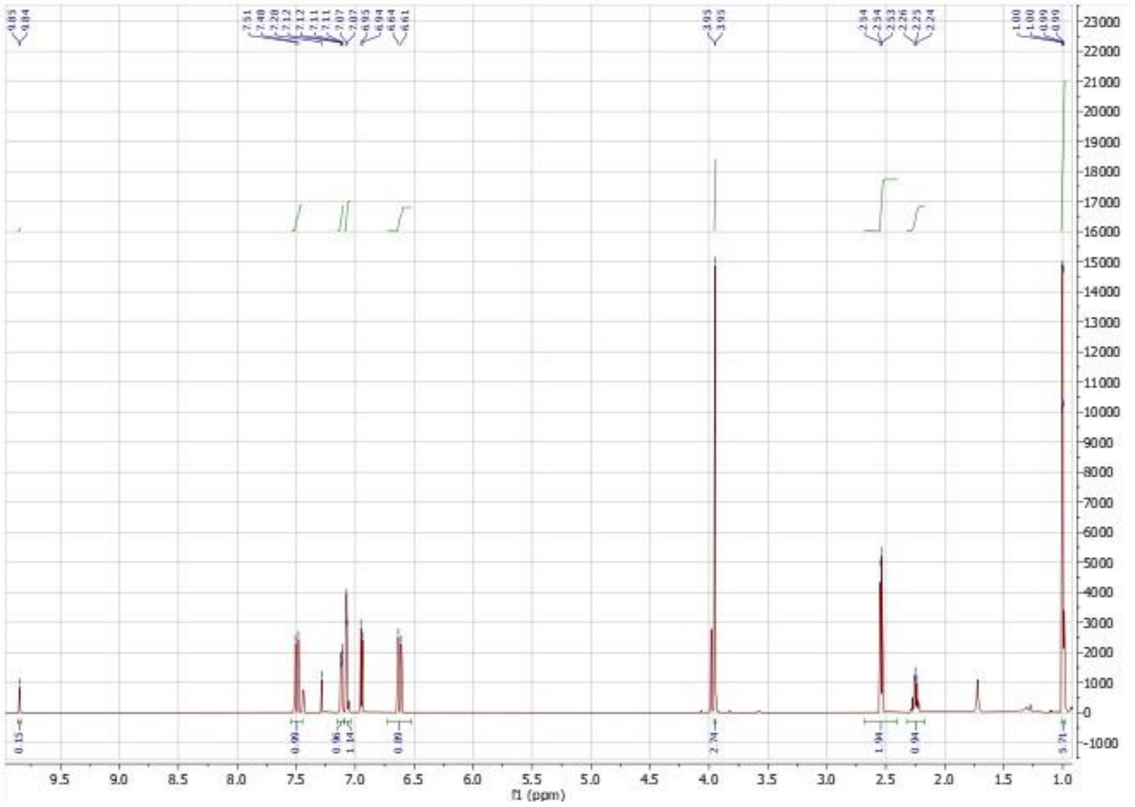

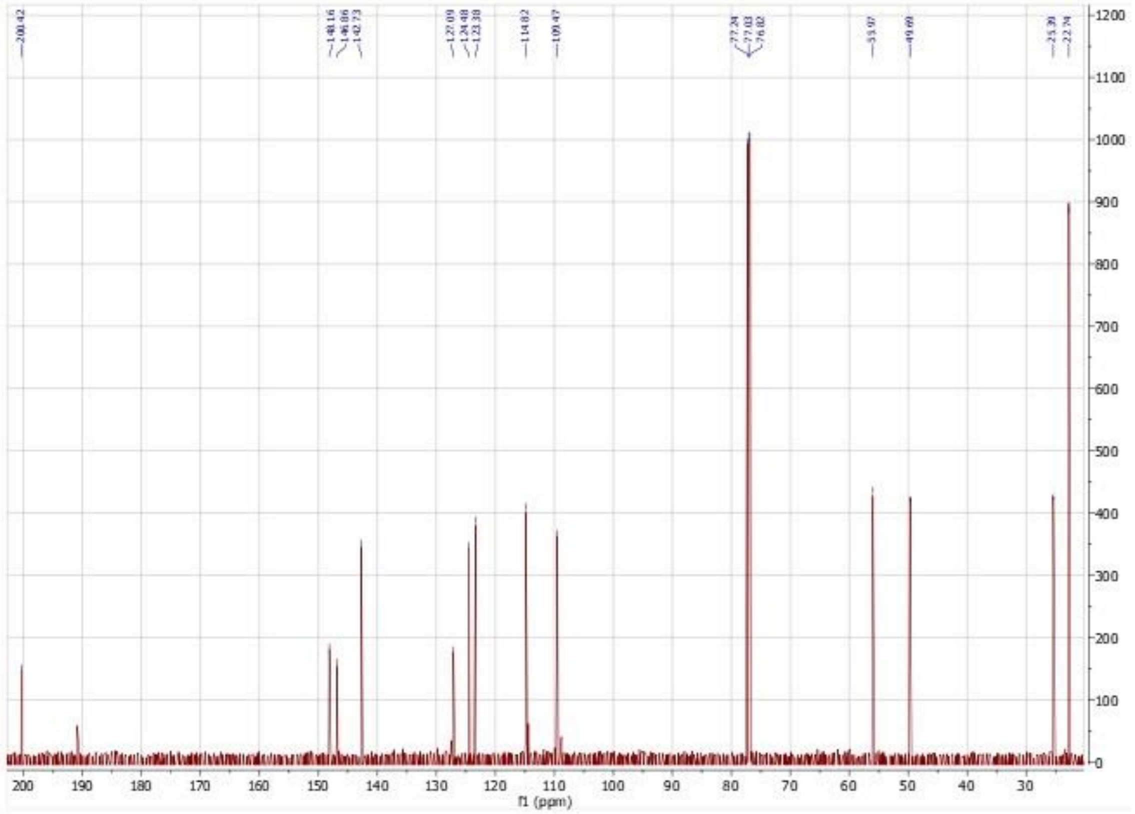

(*E*)-3-(4-hydroxy-3-methoxyphenyl)-1-phenylprop-2-en-1-one (6)

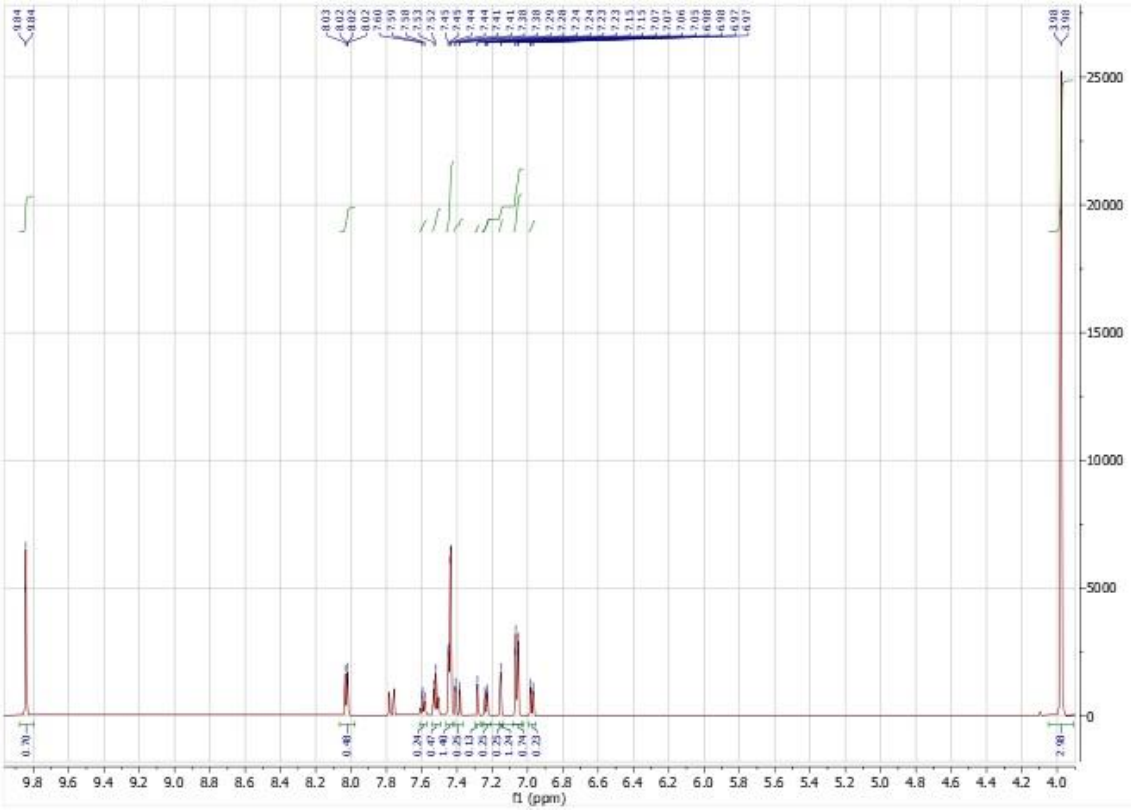

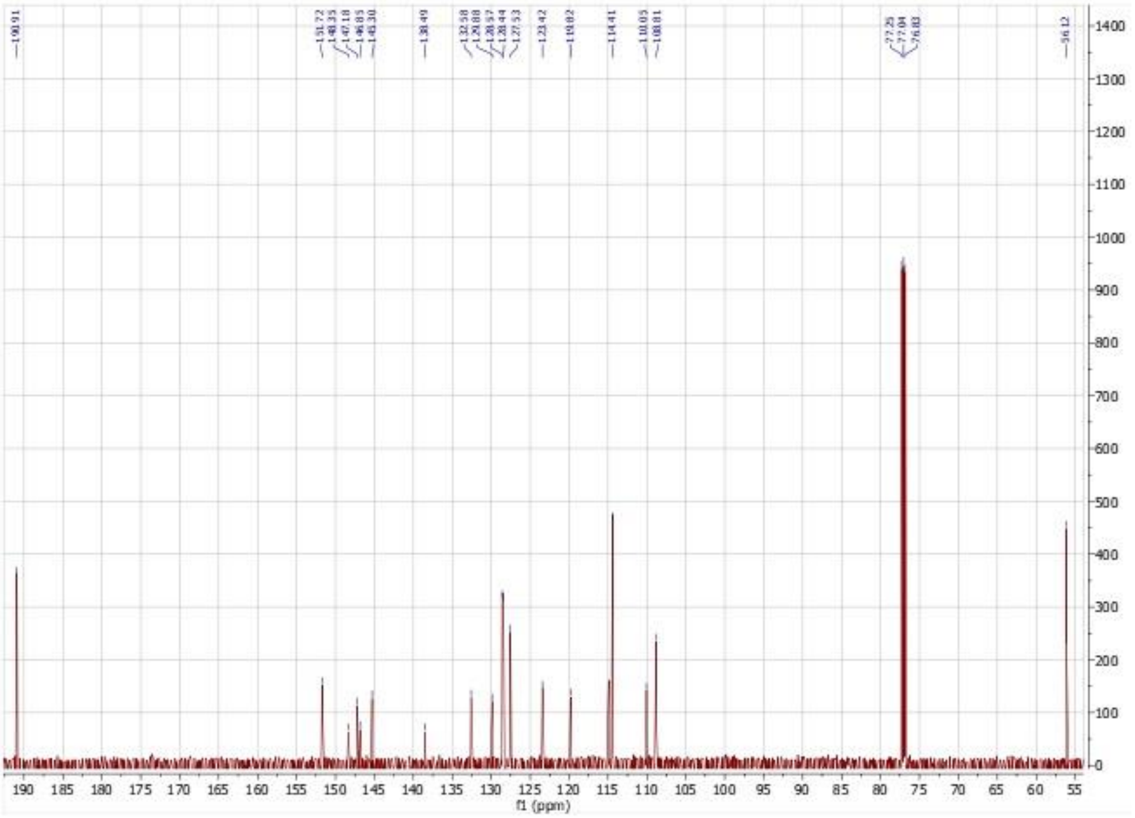

(*E*)-1-(4-bromophenyl)-3-(4-hydroxy-3-methoxyphenyl)prop-2-en-1-one (7)

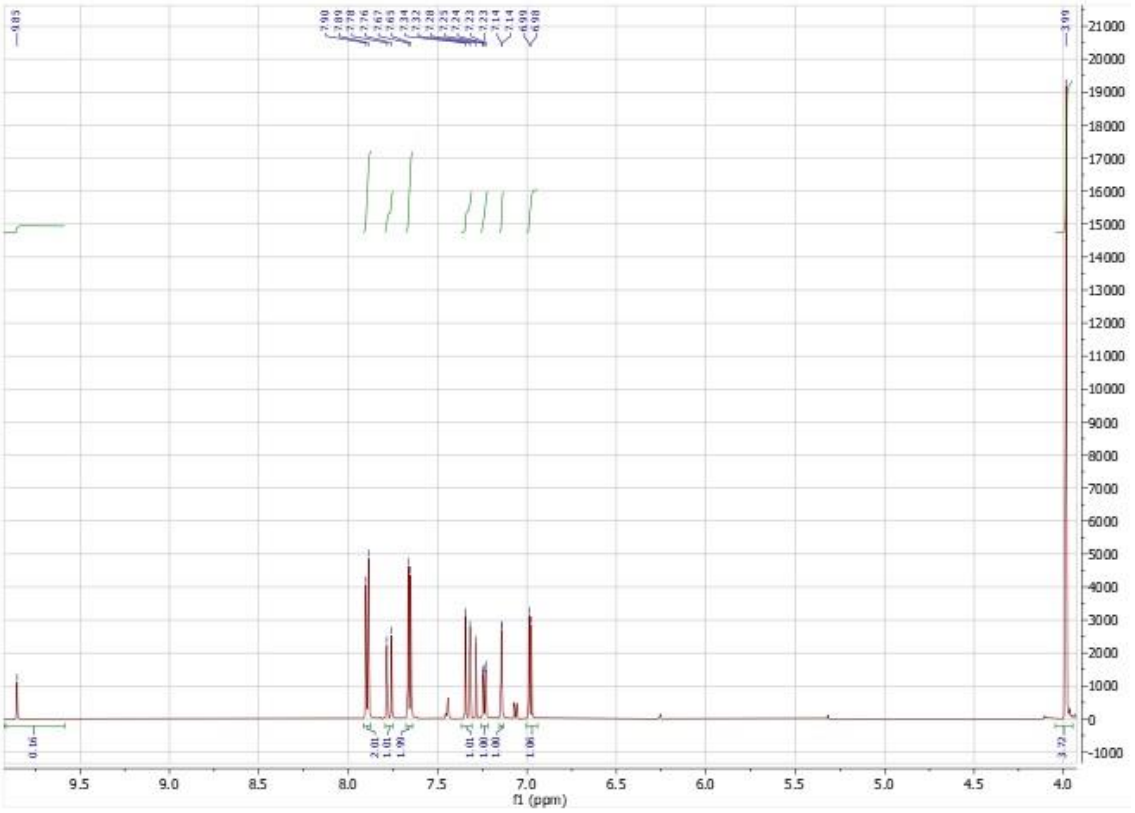

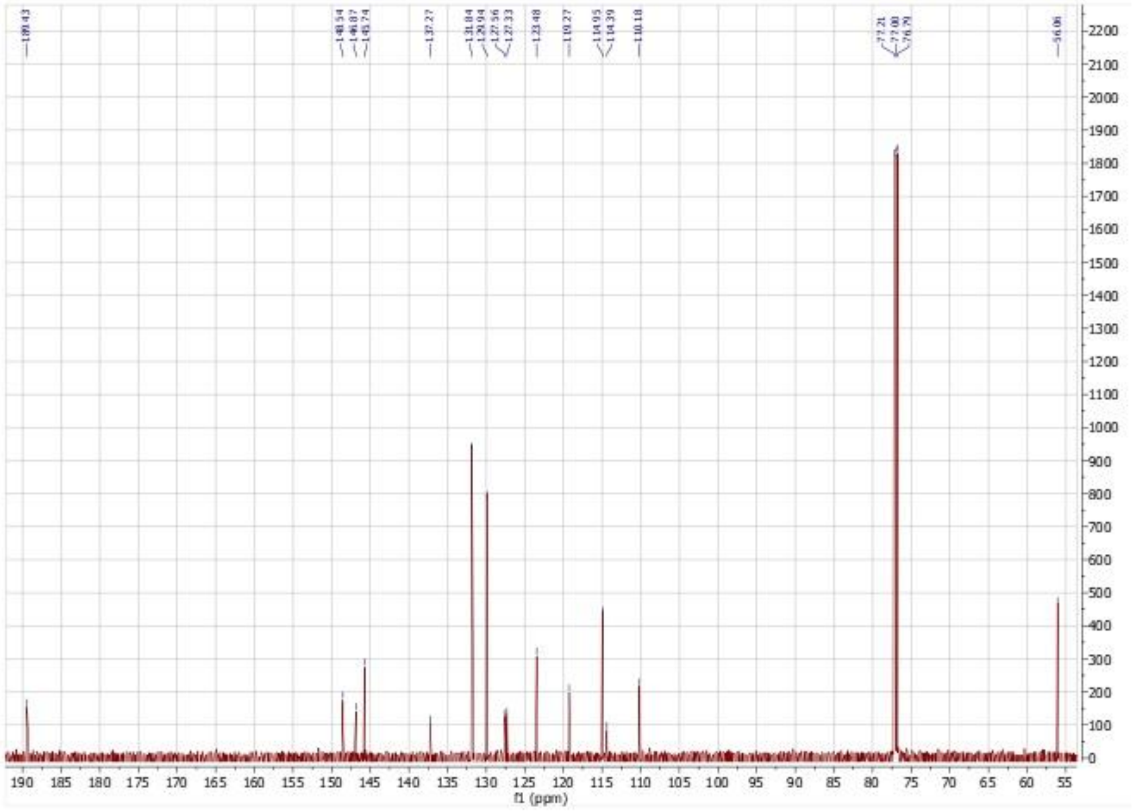

Supplement: Supplemental Material [file IENZ_A_1982933_SM1522.pdf]
